# PARP-inhibition reprograms macrophages toward an anti-tumor phenotype

**DOI:** 10.1016/j.celrep.2022.111462

**Published:** 2022-10-11

**Authors:** Lin Wang, Dan Wang, Olmo Sonzogni, Shizhong Ke, Qi Wang, Abhishek Thavamani, Felipe Batalini, Sylwia A. Stopka, Michael S. Regan, Steven Vandal, Shengya Tian, Jocelin Pinto, Andrew M. Cyr, Vanessa C. Bret-Mounet, Gerard Baquer, Hans P. Eikesdal, Min Yuan, John M. Asara, Yujing J. Heng, Peter Bai, Nathalie Y.R. Agar, Gerburg M. Wulf

**Affiliations:** 1 Cancer Center and Cancer Research Institute, Beth Israel Deaconess Medical Center and Department of Medicine, Harvard Medical School, Boston, MA, USA; 2 Otorhinolaryngology Hospital, The First Affiliated Hospital, Sun Yat-sen University, Guangzhou, Guangdong, China; 3 Department of Neurosurgery, Brigham and Women’s Hospital, Harvard Medical School, Boston, MA, USA; 4 Department of Radiology, Brigham and Women’s Hospital, Harvard Medical School, Boston, MA, USA; 5 Cancer Center and Cancer Research Institute, Beth Israel Deaconess Medical Center and Department of Pathology, Harvard Medical School, Boston, MA, USA; 6 Department of Oncology, Haukeland University Hospital, Bergen, Norway; 7 K.G. Jebsen Center for Genome-Directed Cancer Therapy, Department of Clinical Science, University of Bergen, Bergen, Norway; 8 Division of Signal Transduction, Beth Israel Deaconess Medical Center and Department of Medicine, Harvard Medical School, Boston, MA, USA; 9 Department of Medical Chemistry, Faculty of Medicine, University of Debrecen, Debrecen 4032, Hungary; 10 MTA-DE Lendület Laboratory of Cellular Metabolism, Debrecen 4032, Hungary; 11 Research Center for Molecular Medicine, Faculty of Medicine, University of Debrecen, Debrecen 4032, Hungary; 12 MTAMTA-DE Cell Biology and Signaling Research Group ELKH, Debrecen 4032, Hungary; 13 Hungary-DE Cell Biology and Signaling Research Group ELKH, Debrecen 4032, Hungary; 14 Department of Cancer Biology, Dana-Farber Cancer Institute, Boston, MA, USA; 15 These authors contributed equally; 16 Lead contact

## Abstract

Poly(ADP)ribosylation inhibitors (PARP*i*s) are toxic to cancer cells with homologous recombination (HR) deficiency but not to HR-proficient cells in the tumor microenvironment (TME), including tumor-associated macrophages (TAMs). As TAMs can promote or inhibit tumor growth, we set out to examine the effects of PARP inhibition on TAMs in BRCA1-related breast cancer (BC). The PARP*i* olaparib causes reprogramming of TAMs toward higher cytotoxicity and phagocytosis. A PARP*i*-related surge in NAD+ increases glycolysis, blunts oxidative phosphorylation, and induces reverse mitochondrial electron transport (RET) with an increase in reactive oxygen species (ROS) and transcriptional reprogramming. This reprogramming occurs in the absence or presence of PARP1 or PARP2 and is partially recapitulated by addition of NAD derivative methyl-nicotinamide (MNA). *In vivo* and *ex vivo*, the effect of olaparib on TAMs contributes to the anti-tumor efficacy of the PARP*i*. *In vivo* blockade of the “don’t-eat-me signal” with CD47 antibodies in combination with olaparib improves outcomes in a BRCA1-related BC model.

## In brief

Wang et al. demonstrate that a PARP inhibitor used to treat breast tumors with homologous recombination (HR) deficiency reprograms the HR-proficient macrophages in the tumor microenvironment toward an anti-tumor phenotype. Blocking the “don’t-eat-me” signal on tumor cells with anti-CD47 antibodies enhances the therapeutic efficacy of olaparib in a mouse model.

## Graphical Abstract

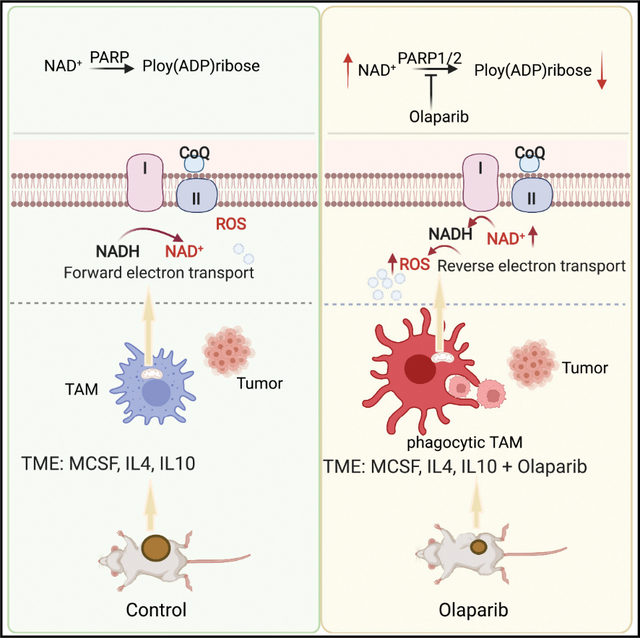

## INTRODUCTION

PARP inhibition has emerged as an effective and well-tolerated treatment for epithelial cancers with deficiencies in homologous recombination (HR deficiency), most prominently tumors with loss of function in BRCA1/2. A striking observation in clinical trials has been that despite a similar spectrum of oncogenic mutations, the efficacy of PARP inhibition is different in tumors depending on the tissue of origin: in the first phase 2 study of olaparib, the rate of partial remissions was 46% and 24% in patients with ovarian cancer with and without a BRCA1/2 mutation, respectively, while in the same study, none of the patients with breast cancer (BC), including 10 BRCA1/2 mutation carriers, had any clinical benefit (Gelmon et al., 2011). Subsequently, more extensive studies confirmed that even in heavily pretreated BRCA1/2 mutation carriers with ovarian cancer, olaparib delivered a median duration of response of 8 months (Domchek et al., 2016; Matulonis et al., 2016), while a similar benefit was seen in patients with BC with a BRCA1/2 mutation only in the sensitive first-line setting (Robson et al., 2017). Hence, despite a similar spectrum of predisposing BRCA1/2 mutations, patient outcomes differ depending on the tissue of origin. Cell line sensitivity assays, however, suggest similar sensitivity to PARP inhibition irrespective of the tissue of origin (Figure S1A). These observations, similar *in vitro* sensitivity to PARP inhibition of breast and ovarian cancer cell lines, but different clinical efficacy of PARP inhibition in patients with ovarian cancer and BC, allow for the possibility that factors independent of the primary tumor cell type contribute to the effectiveness of PARP inhibition in BRCA1/2-mutant tumors, such as differences in the composition of the tumor microenvironment (TME) and specifically the innate immune system (Kubli et al., 2019; Mehta et al., 2021; Morse et al., 2019).

We had previously found that treatment outcomes of olaparib in a mouse model of BRCA1-related BC depended on the TME: median survival was greatest when the tumor was implanted in syngeneic, immune-competent animals, significantly lowered when CD8 cells were depleted, and further decreased when the same tumor was treated as an allogeneic transplant in a SCID/beige host, suggesting that TME components beyond CD8 cells contribute to olaparib’s efficacy (Pantelidou et al., 2019). In the same study, we also showed that the largest component of the TME was myeloid-derived cells and specifically macrophages. As olaparib is not toxic to cells proficient in HR, such as the cells of the TME, and given the high abundance of macrophages in the TME of BRCA1/2-mutant cancers, we examined how PARP inhibition modulates tumor macrophage function. We found that PARP inhibition leads to reprogramming the metabolism of tumor-associated macrophages (TAMs), ultimately contributing to a favorable outcome.

## RESULTS

### Macrophages contribute to the therapeutic efficacy of PARP*i*s

**Figure 1.**
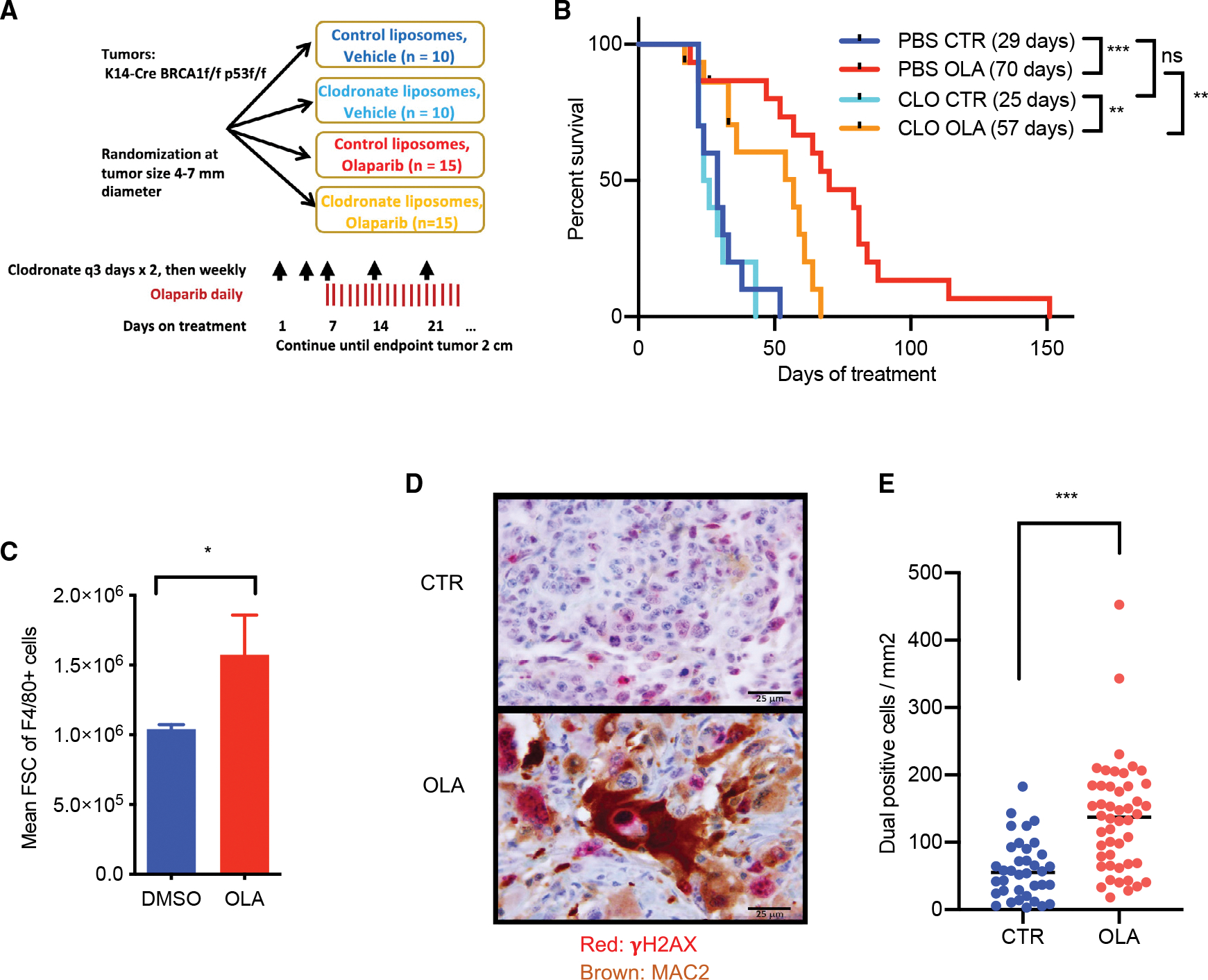
Olaparib augments anti-tumor macrophage activity (A and B) Macrophage ablation decreases the efficacy of olaparib. (A) Experimental design. K14-Cre BRCA1f/f p53 f/f tumors (hereafter referred to as K14 tumors) were implanted in syngeneic mice, and mice were randomized when the tumors reached 4–7 mm in diameter. Clodronate liposomes or PBS control liposomes were administered twice a week for the first week (100 μL/mouse), followed by once a week; olaparib treatment was daily. (B) Survival statistics, mice stratified according to treatment, log rank test. The preset endpoint was a tumor diameter of 20 mm. In brackets is shown the median survival for each cohort in days from the start of treatment. (C–E) Macrophages in olaparib-treated tumors are large and highly phagocytic. (C) Reanalysis of forward scatter signal (FSC) as a readout for cell volume for F4/80^+^ cells as published (Pantelidou et al., 2019; n = 5 mice for olaparib and control each). Tumors harvested from mice treated with olaparib and corresponding controls were dissociated, stained with F4/80, and subjected to flow cytometry. Data are presented as mean ± SD. Significance was determined by an unpaired t test. (D and E) K14 tumors were implanted in syngeneic mice, randomized when the tumors reached 4–7 mm in diameter, and euthanized after 10 days, and olaparib-treated (n = 8) and control (n = 6) tumors were subjected to dual-stain immunohistochemistry with γH2AX (red) and MAC2 (brown), scale bar = 25μm (D), and in each tumor, dual-positive cells were counted in six random fields (E). Data are presented as mean ± SD. Significance was determined by the Mann Whitney test, ***p < 0.001.

To understand the role that TAMs play in the efficacy of the poly(ADP)ribosylation inhibitor (PARP*i*) olaparib in an immune-competent host, we treated a cohort of tumor-bearing mice with and without ablation of TAMs. Ablation of macrophages was achieved with the liposomal bisphosphonate clodronate (Zeisberger et al., 2006). Cohorts of tumor-bearing mice were created using primary tumors isolated from a K14-Cre BRCA1f/fp53f/f mouse model (Liu et al., 2007; Rottenberg et al., 2007) and transplanted into syngeneic immune-competent recipients. Once tumors reached a size of 4–7 mm in diameter, mice were randomized to macrophage ablation with clodronate and olaparib treatment according to the scheme in Figure 1A. As expected (Zeisberger et al., 2006), clodronate was highly effective to ablate TAMs, which were reduced by greater than 60% (Figures S1B–S1D). We found that clodronate as a single agent did not affect the growth of these tumors (median tumor-specific survival was 25 days with clodronate versus 29 with control liposomes) (Figures 1B and S1E). Tumor burden at study entry was evenly balanced among cohorts (Figure S1F). Olaparib was, as expected, highly effective (Pantelidou et al., 2019; Rottenberg et al., 2007) and improved median survival to 70 days. However, in combination with clodronate, median survival dropped to 59 days, indicating that the ablation of macrophages had a negative impact on the efficacy of olaparib (Figures 1B and S1E). A negative effect on olaparib outcomes was not seen when the same tumor was treated with clodronate in an immune-compromised SCID/beige host (Figures S1G and S1H).

The improvement of olaparib outcomes in immune-competent mice was a surprising finding, as ablation of TAMs with clodronate, in general, has been reported to slow tumor growth (Carron et al., 2017; Zeisberger et al., 2006). Our finding of worse treatment outcomes when olaparib was combined with clodronate in an immune-competent host, however, suggested that in the specific setting of PARP inhibition, TAMs did not promote tumor growth but contributed to the anti-tumor response with olaparib.

### PARP inhibition induces large macrophages with the ability to kill and phagocytose cancer cells

We examined if the function or morphology of macrophages changed during treatments with olaparib and observed that macrophages in olaparib-treated tumors were larger than in control tumors upon reanalysis of the forward scatter signal in our prior flow cytometry data (Pantelidou et al., 2019) (Figure 1C). As macrophages grow in size when they phagocytose, we determined the phagocytic index, i.e., the number of macrophages that had engulfed γH2-AX-positive tumor cells (Figure 1D), reasoning that γH2-AX-positive nuclei in macrophages would be derived from tumor cells with BRCA1 loss given that the host macrophages are BRCA1 proficient. Macrophages were stained with antibodies against galectin-3 (Mac-2), a pan-macrophage marker functionally important for phagocytosis (Sano et al., 2003). The number of phagocytic macrophages in olaparib-treated tumors was twice as high as in the vehicle control (Figure 1E).

**Figure 2.**
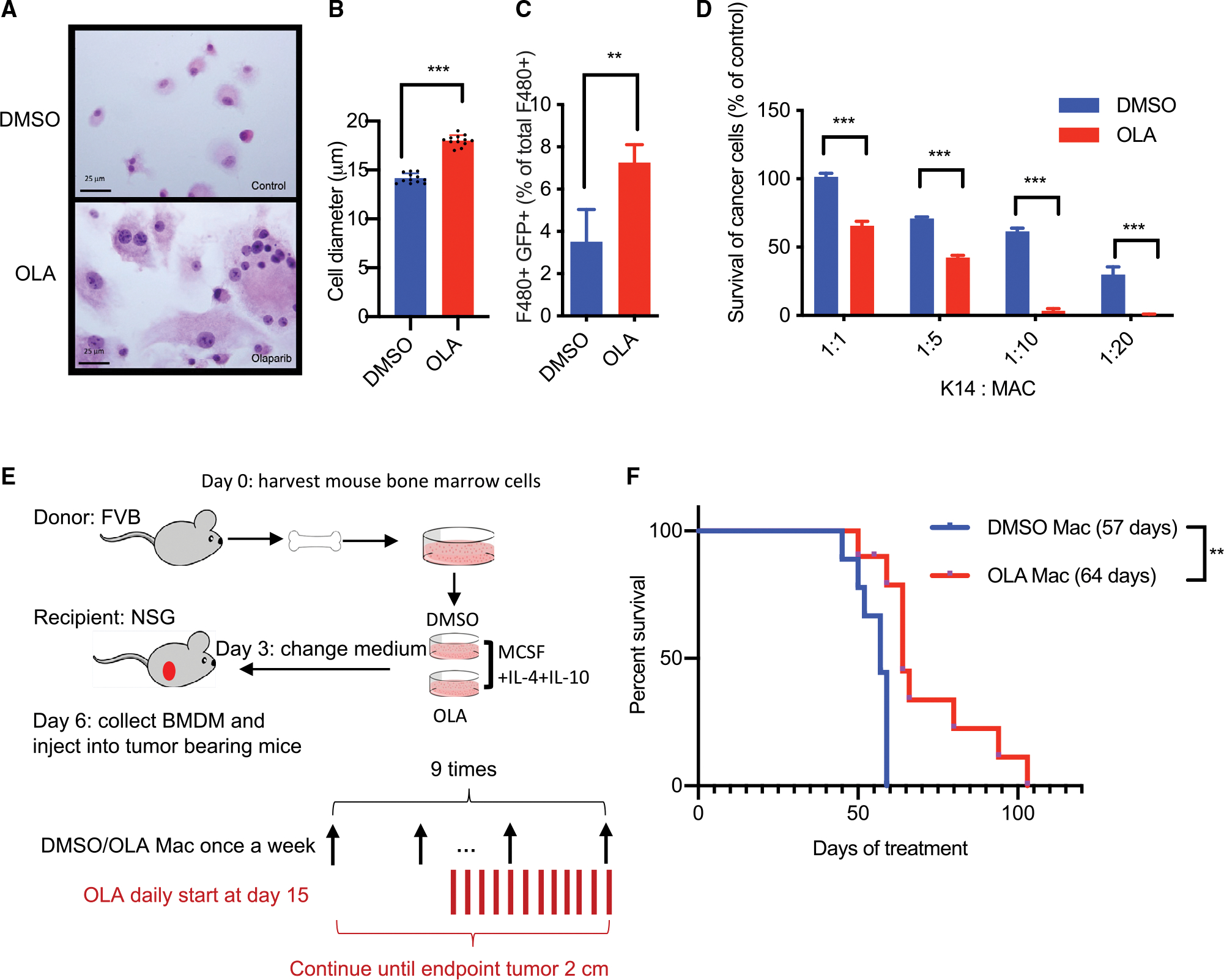
Olaparib directly reprograms alternatively activated macrophages toward an anti-tumor activity (A and B) Mouse bone marrow cells (6 donor mice) were cultured on culture slides in the presence of IL-4, IL-10, and M-CSF and olaparib or DMSO. Cells were stained with hematoxylin and eosin on day 6, scale bar = 25μm (A). Cell diameter measured by Cellometer, 4 replicates per group, 3 independent experiments; data are presented as mean ± SD, unpaired t test (B). (C) Increased phagocytosis upon olaparib treatment. Mouse bone marrow cells were cultured as in (A) but in 10 cm plates, harvested, and co-cultured with GFP-expressing K14-BRCA1f/f p53f/f cancer cells (hereafter referred to as K14-GFP cells) for 4 h at a ratio of 1:1 and stained with F4/80 antibodies. The percentage of dual-positive cells was determined by flow cytometry (4 donor mice, 4 replicates per group, data presented as mean ± SD). (D) Mouse bone marrow cells were treated as in (A) and then co-cultured with 5,000 K14-GFP cells for 24 h at ratios as indicated (5 replicates per group, data presented as mean ± SD). Bioluminescence assay (hereafter referred to as BLI assay) was performed to detect the fraction of surviving cancer cells. (E and F) Adoptive transfer of macrophages precultured *ex vivo* into NSG mice (10 tumor-bearing mice/arm). Bone marrow from FVB donor mice (5 donor mice per time point) was cultured as in (A) in the absence or presence of olaparib or vehicle for 6 days. Bone marrow-derived macrophages (BMDMs) were intratumorally injected in recipient NSG mice weekly 9 times (5 × 10^5^/mouse), and olaparib was administered daily starting at day 15 after the first macrophage injection (E). Survival according to Kaplan Meier after adoptive transfer of macrophages pretreated with olaparib or DMSO *ex vivo*, and median survival in days is shown in parentheses (F). Cell culture data are presented as mean ± SD. Significance was determined by two-way ANOVA, ***p < 0.001. *In vivo* data were subjected to the log rank test.

To discern if the anti-tumor macrophage activity in olaparib-treated tumors directly affects olaparib on macrophages or the indirect result of olaparib-induced apoptosis of tumor cells, we examined the effects of olaparib on macrophage morphology and function *ex vivo* in cultures of bone marrow-derived macrophages (BMDMs). We differentiated these BMDMs for 6 days toward a pro-tumor (M2) phenotype using M-CSF, interleukin-4 (IL-4), and IL-10 (Mia et al., 2014) and examined the effect of olaparib on these M2 macrophages (Figure S2A). We found that in the presence of olaparib, these *ex vivo*-differentiated macrophages grew substantially in size, from a mean diameter of 14 to 18 μm, resulting in a doubling of their volume and forming clusters of giant cells, with expansive cytoplasm and ingested cell debris (Figures 2A and 2B). Olaparib-treated macrophages, but not control macrophages, were able to ingest GFP-labeled K14-Cre BRCA1f/fp53f/f BC cells (Figures 2C and S2B), indicative of a functional change toward an anti-tumor phenotype. Moreover, the *ex vivo*-differentiated, olaparib-treated macrophages had a greater ability to kill K14-Cre BRCA1f/fp53f/f BC cells than their controls (Figure 2D), and that required at least 4 days of *ex vivo* differentiation to develop (Figure S2B). These data support a direct effect of the PARP*i* on differentiated M2 macrophages that results in reprogramming toward tumor cell killing and phagocytosis.

To understand if this direct reprogramming of macrophages by PARP inhibition contributes to the anti-tumor response elicited by olaparib *in vivo*, we introduced *ex vivo*-differentiated macrophages into tumors *in vivo* (Figures 2E, 2F, S2C, and S2D). Using Q-tracker to label macrophages *ex vivo* prior to injection, we determined that these macrophages persist for as long as 7 days after harvesting, dissociating, and gating on live CD45/F4/80-positive cells (Figure S2E). As hosts, we used tumor-bearing NSG mice incapable of developing anti-tumor macrophages themselves (Shultz et al., 2000). Tumor-bearing mice received intratumoral injections of macrophages once a week and olaparib daily. Treatments were stopped when all mice in the control cohort had reached the endpoint (Figure 2E). We found that olaparib-pretreated macrophages significantly delayed and slowed the progression of BRCA1-deficient breast tumors (Figure 2F). These data confirm *in vivo* that olaparib-induced reprogramming of macrophages contributes to a biologically significant degree to the anti-tumor activity of the PARP*i*.

### PARP inhibition reprograms macrophages toward a functionally inflammatory state

**Figure 3.**
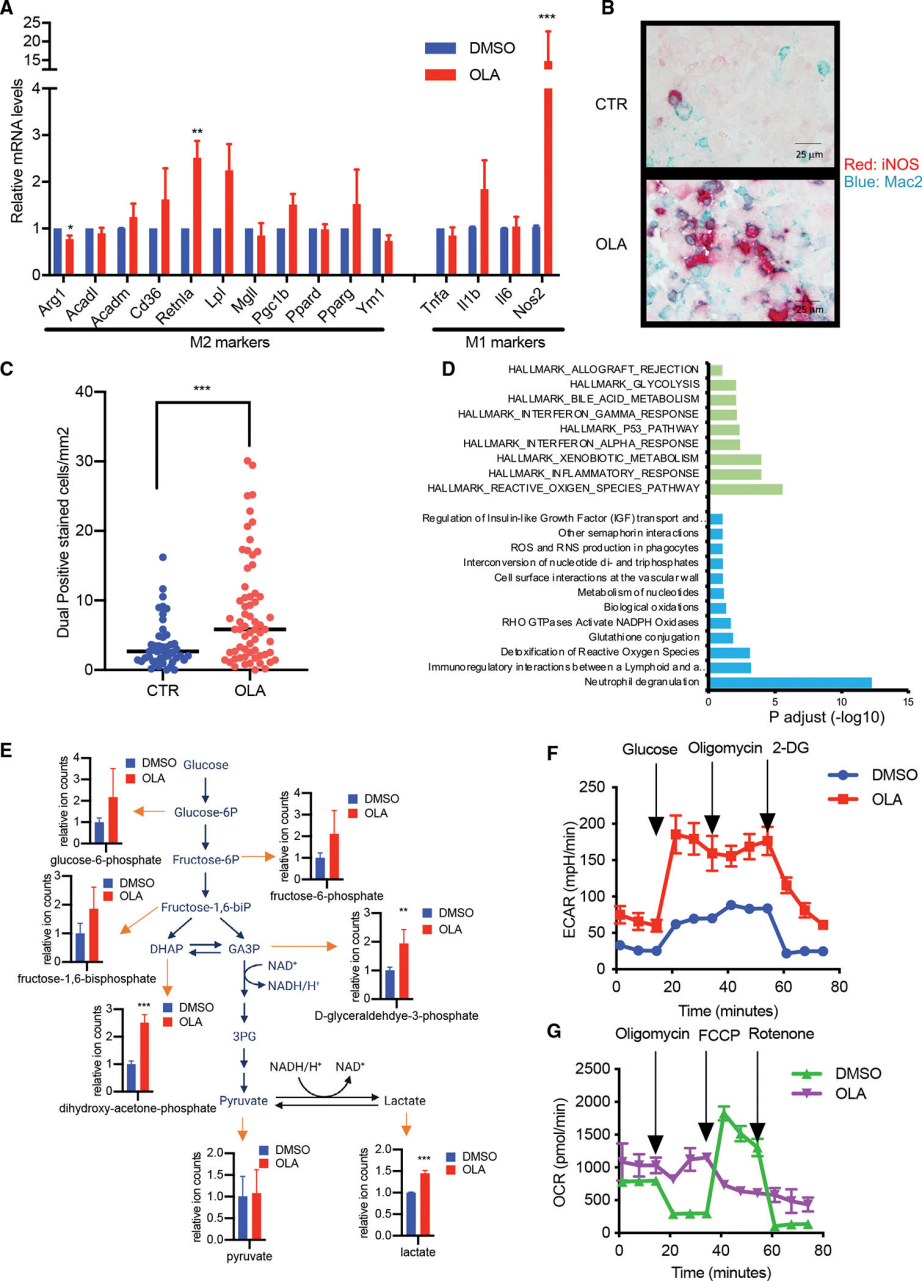
PARP inhibition induces a pro-inflammatory macrophage phenotype (A and D–G) Mouse bone marrow cells were cultured as indicated in the absence or presence of olaparib or DMSO for 6 days (hereafter referred to as BMDMs), 3 donor mice for each experiment. (A) Total mRNAs were extracted and subjected to quantitative real-time PCR. Mean ± SD of three replicates, representative of three separate experiments. (B and C) K14 tumors from mice treated for 10 days with olaparib were subjected to dual-stain IHC with iNOS (red) and MAC2 (blue), scale bar = 25μm (B), and 8 fields of each slide from 6 control tumor-bearing mice and 8 olaparib treated tumor-bearing mice were counted for double positive cells. Mean ± SD, Mann Whitney test (C). (D) RNA from macrophages from 4 donor mice per experiment was differentiated *ex vivo* in the absence or presence of olaparib (quadruplicates for each group) and subjected to RNA-seq analysis. Pathway analysis of 500 top up-regulated genes. Green bars represent up-regulated genes in Hallmark pathways, and blue bars represent up-regulated genes in Reactome pathways. (E) Polar metabolites were extracted from BMDMs from 4 donor mice per experiment, cultured as indicated, and analyzed by mass spectrometry. Data presented as mean ± SD of 4 replicates, and comparisons were made using an unpaired t test. (F and G) Glycolytic flux is up-regulated and oxygen consumption down-regulated in olaparib-treated macrophages. BMDMs were harvested and 1 × 10^5^/well seeded in a Seahorse assay 24-well plate and cultured overnight. Extracellular acidification (F) and oxygen consumption rates (G) were determined using a Seahorse analyzer (each time point represents the mean ± SD of 5–6 cell culture replicates of bone marrow from 3 donor mice per experiment, representative of two individual experiments).

As olaparib-induced reprogramming of macrophages occurred gradually over 6 days of culture (Figure S2B), we tested for transcriptional reprogramming. Characterization of olaparib-treated macrophages with quantitative RT-PCR revealed a 15-fold up-regulation of inducible nitric oxide synthase (iNOS) (Figures 3A and S3A), while overall protein levels for arginase did not significantly change (Figure S3A). Dual-stain immunohistochemistry (IHC) of MAC2 and iNOS in control and olaparib-treated tumors confirmed a strong induction of iNOS expression by day 10 of treatment, consistent with the generation of an oxidative environment in which iNOS contributes to the oxidative burst of macrophages and their anti-tumor activity (Figures 3B and 3C). In contrast, the expression profile for CD86 and CD206 did not significantly change (Figures S3B and S3C). These observations were replicated in a different K14-Cre BRCA1f/fp53f/f tumor model on the FVB/N background (Figure S3D), and, interestingly, a trend toward such reprogramming was observed in a third model with BRCA1 proficiency (K14-Cre BRCA1wt/wtp53f/wt; Figure S3E). Unbiased profiling of control and olaparib-treated macrophages with RNA sequencing (RNA-seq) confirmed olaparib-induced reprogramming of macrophages toward the production of reactive oxygen and nitrogen species, phagocytosis, and glycolysis (Figures 3D and S3F). To understand if these observations in murine tumors translated to humans, we stained pre- and post-treatment sections from tumors of two women who participated in the PETREMAC study (ClinicalTrials.gov: NCT02624973) on neo-adjuvant olaparib for triple-negative BC (Eikesdal et al., 2019). Although we were only able to obtain two matched pre- and post-olaparib biopsies from patients, the increased density of iNOS-positive macrophages in these patients is consistent with our observations in mice (Figure S3G). In summary, we observed changes in olaparib-treated tumor macrophages that suggest a shift toward a pro-inflammatory and anti-tumor function.

### PARP inhibition induces reverse electron transport (RET) leading to enhanced reactive oxygen species (ROS) production

To determine if there was a shift in metabolism that corresponds to the observed shift in function, we examined polar metabolites in PARP*i*-treated macrophages and their controls (Figures 3E and S3H). We found an increase in glycolytic metabolites (Figure 3E) as well as a trend toward increased nitrogen processing through the urea cycle (Figure S3H). Consistently, measurement of extracellular acidification (ECAR) in a Seahorse analyzer revealed increased glucose avidity that could, however, not be further increased with the extinction of the TCA cycle with oligomycin, in response to PARP*i* treatment, indicative of greater reliance on glycolytic flux after PARP*i* treatment (Figures 3F and S3I). In control macrophages, the oxygen consumption rate (OCR) declined after inhibition of ATP synthase with oligomycin and was greatly rescued with the uncoupling protonophore FCCP, followed by complete extinction with the complex I inhibitor rotenone (Figures 3G and S3J), indicative of active mitochondrial metabolism. After PARP*i* pretreatment, the baseline OCR of macrophages was slightly higher than in controls but was much less sensitive to oligomycin and not increased by FCCP (Figures 3G and S3J), suggesting that oxygen consumption was not used to drive forward electron transport and ATP production but was used for the generation of ROS, possibly through RET.

**Figure 4.**
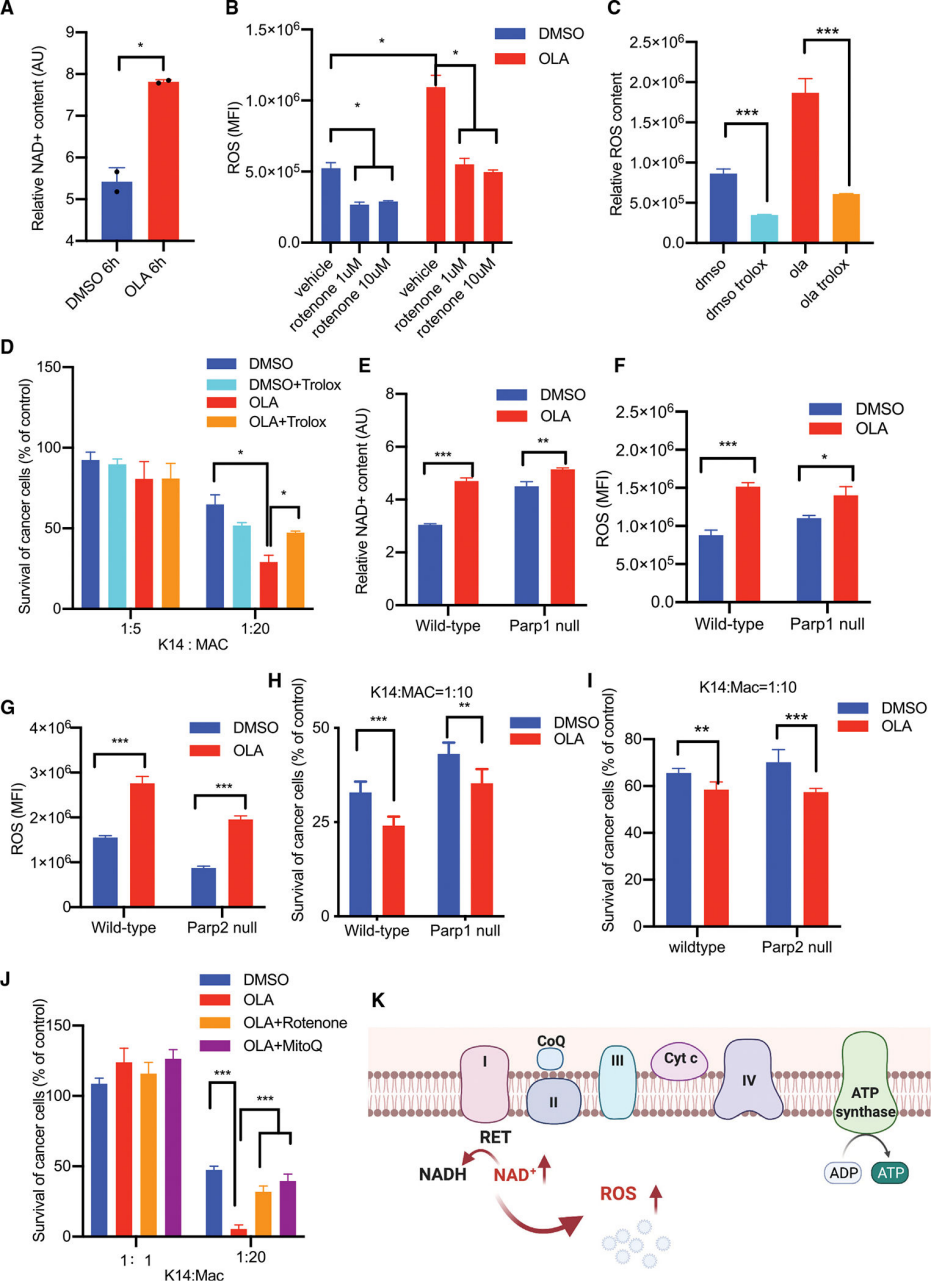
PARP inhibition facilitates ROS production via RET and induces macrophage reprogramming independent of PARP1/2 (A) Acute increase of macrophage NAD levels in response to olaparib. BMDMs were treated with olaparib for 6 h prior to lysis for NAD determination (3 donor mice per experiment, data presented as mean ± SD of duplicate cultures, representative of two independent experiments). (B) ROS production is rotenone sensitive. BMDMs were harvested and suspended in ROS detection buffer in the presence of rotenone as indicated (20 min). Incubation with DCFCA for another 30 min, followed by flow cytometry. (C and D) BMDMs were treated with olaparib alone or together with trolox. (C) Cells were stained with DCFDA, and ROS levels were analyzed by flow cytometry. (D) Cells were co-cultured with K14-GFP cells for 24 h, and BLI assay was performed to evaluate phagocytosis of the BMDMs. (E–I) BMDMs from PARP1/2-null mice and wild-type controls were cultured as in Figure 3A. (E) NAD determination and (F and G) ROS determination. 3 donor mice per experiment in (A)–(G). Data presented as mean ± SD of triplicate cultures. (H and I) Phagocytosis assay (co-culture with K14-GFP cells followed by BLI assay). Data presented as mean ± SD of 5 (H) or 6 (I) replicates obtained from 3 donor mice per experiment, representative of a duplicate experiment. (J) BMDMs were cultured as in Figure 3A with DMSO, olaparib in the absence or presence of rotenone, or MitoQ prior to co-culture with K14-GFP cells as indicated, and analysis of phagocytosis with the BLI assay. Data represent 6 replicates obtained from 3 donor mice, presented as mean ± SD, representative of a duplicate experiment. For all data, significance was determined by the unpaired Student’s t test. *p < 0.05, **p < 0.01, and ****p < 0.0001. (K) Mechanism of ROS via reverse electron transport (RET) induced by accumulation of the PARP substrate NAD.

RET occurs when the proton motive force across the inner mitochondrial membrane is used to drive electrons in the reverse direction through the electron transport chain to reduce NAD (Diskin and Palsson-McDermott, 2018; Mills et al., 2016; Robb et al., 2018). We hypothesized that PARP inhibition could acutely lead to an increase in the PARP substrate NAD, which then would be available for RET. PARP enzymes use NAD as a substrate for poly-ADP(ribosyl)ation (PARylation) of itself and its target proteins. We confirmed that BMDMs expressed PARP1/2 and that olaparib did inhibit PARylation in these cultures (Figures S4A and S4B), resulting in rapid accumulation of the PARP substrate NAD+ (Figure 4A), which is available as an electron acceptor in RET.

To understand if RET was mechanistically linked to increased phagocytosis (Figures 1D, 1E, and 2A–2D) and baseline OCR (Figure 3G), we examined ROS production upon treatment with olaparib. We found a more than 2-fold increase in ROS production in olaparib-treated macrophages (Figures 4B and S4C–S4I). ROS production was rotenone sensitive, consistent with RET, as rotenone prevents co-enzyme Q (CoQ) from transferring electrons back to complex I (CI) and specifically reduces ROS production that results from RET (Mills et al., 2016; Robb et al., 2018) (Figure 4B). Consistent with a process of metabolic reprogramming, the increase in ROS production upon PARP inhibition occurred gradually and was detectable within 48–96 h of PARP inhibition (Figures S4C–S4I). This increase in ROS production was supported by a higher MITOsox stain (Figures S4J and S4K) in olaparib-treated macrophages, while the mitochondrial mass, as assessed by MITOtracker, was not significantly changed (Figures S4L and S4M). With an alternative ROS scavenger, the vitamin E analog trolox, olaparib-induced ROS production was similarly suppressed (Figure 4C), and the anti-tumor activity of induced macrophages was severely reduced (Figure 4D). In summary, we found that the rapid increase in NAD caused by PARP inhibition was accompanied by a stark increase in ROS production from RET, which is characterized by a reduction of NAD to generate ROS, and subsequent increased phagocytic activity.

### Olaparib-induced macrophage reprogramming occurs in the absence of PARP1 or PARP2

To determine if reprogramming was the result of suppression of specific target protein PARylation or of accumulation of intracellular NAD, the substrate used by PARP1 and PARP2, we compared the effects of olaparib on PARP1- or PARP2-null and wild-type (WT) control macrophages. We reasoned that if reprogramming was indeed the consequence of NAD accumulation in response to PARP inhibition, it should occur at least to some extent in the absence of PARP1 or PARP2 because olaparib, although predominantly an inhibitor of PARP1 (Figure S4A), also inhibits PARP2 (Figure S4B) and, to a lesser extent, other PARP enzymes, and hence with genetic ablation of a single PARP enzyme, some NAD accumulation would still be expected. On the other hand, if specific PARylation by either PARP1 or PARP2 was required, phenotypes should differ between PARP1 and PARP2 knockout (KO) macrophages. As expected in the absence of the NAD-consuming PARP1, baseline NAD levels were higher in PARP1-null-derived macrophages than in the corresponding WT controls (Figure 4E). This corresponded to higher baseline ROS levels in both PARP1- (Figure 4F) and PARP2-null (Figure 4G) macrophages. But a significant increase of NAD was still observed upon olaparib treatment, consistent with inhibition of PARP enzymes other than PARP1 (Figure 4E). Consequently, olaparib treatment increased ROS levels in all three macrophage populations, WT, PARP1-null (Figure 4F), or PARP2- null (Figure 4G). Similarly, the PARP*i* induced an increase in cell size (Figures S4N and S4O) in iNOS mRNA (Figures S4P and S4Q) and, finally, the ability to kill tumor cells *ex vivo* (Figures 4H and 4I) in PARP1- or PARP2-null macrophages. These data show that the ability to induce phagocytic activity did not strictly depend on specific inhibition of PARP1- or PARP2-mediated PARylation, and they allow for the possibility that accumulation of the common PARP substrate NAD mediates the effect.

If increased ROS as a result of RET with reduction of NAD was the cause of the increased anti-tumor activity of PARP*i*-treated macrophages, then the anti-tumor activity of macrophages should be abolished by inhibitors of RET. To specifically examine the effect of inhibition of RET on the anti-tumor activity of macrophages, we employed treatment with either rotenone, which blocks the ubiquinone reduction site of complex I (IQ site) and prevents CoQ from transferring electrons back to CI (Mills et al., 2016; Robb et al., 2018), or MitoQ, which increases ROS under conditions of forward electron transport (FET) and reduces ROS production under conditions of RET (O’Malley et al., 2006). We found that in addition to trolox (Figure 4D), rotenone and MitoQ diminished the olaparib-induced anti-tumor activity of macrophages (Figure 4J), consistent with ROS as a result of RET causing olaparib-induced reprogramming of macrophages (Figure 4K).

### The NAD derivative methyl-nicotinamide induces ROS production and phagocytic activity

**Figure 5.**
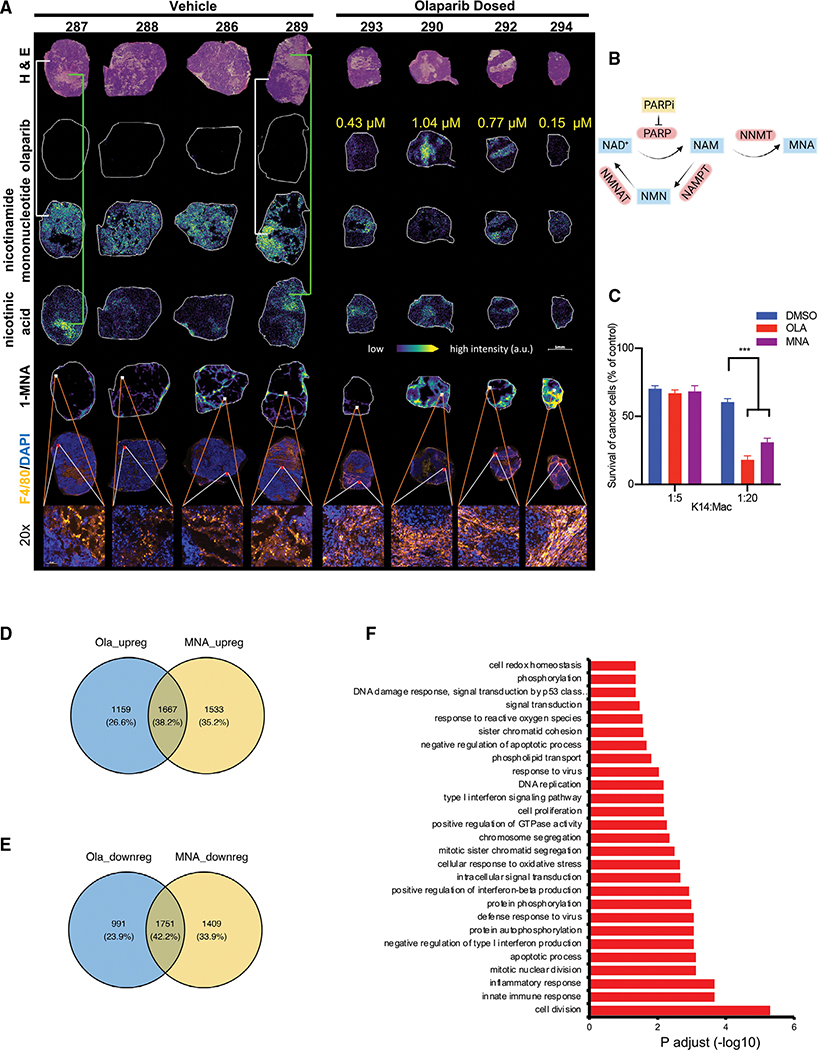
PARP inhibition leads to a shift in nicotinamide metabolism including accumulation of methyl-nicotinamide (MNA), which can phenocopy olaparib’s effect on BMDMs (A) K14-Cre BRCA1f/fp53f/f tumor-bearing mice were treated with olaparib for 10 days, with 4 tumor-bearing mice per group. Euthanasia 2 h after the last olaparib dose, tumors were immediately snap frozen, and frozen sections were prepared for *in situ* mass spectrometry and immunofluorescence staining. Hematoxylin and eosin (H&E), olaparib levels, and NAD precursors nicotinamide mononucleotide, nicotinic acid, and 1-MNA were analyzed on serial frozen sections by use of matrix-assisted laser desorption ionization mass spectrometry imaging (MALDI-MSI) on 5 μm frozen sections. Brightening color represents the increasing abundance of the measured parameter. F4/80 antibodies (red fluorescence) were used to stain macrophages (6th and 7th row). Red (F4/80) and white (MNA) outlined squares correspond to each other in these serial sections. Scale bar = 5mm. (B) Biosynthesis of MNA. NAM, nicotinamide; NMN, nicotinamide mononucleotide; NMNAT, nicotinamide mononucleotide adenylyl transferase; NAMPT, nicotinamide phosphoribosyltransferase; NNMT, nicotinamide N-methyltransferase. (C) MNA induces a prophagocytic phenotype. Mouse BMDMs were cultured as in Figure 3A with DMSO, olaparib, or MNA and co-cultured with K14-GFP cells, followed by phagocytosis via BLI assay (6 donor mice per experiment, 6 culture replicates per experiment, data represent the mean ± SD of a representative duplicate experiment). For all data, significance was determined by unpaired Student’s t test. *p < 0.05, **p < 0.01, and ****p < 0.0001. (D–F) Overlapping expression profiles induced by olaparib or MNA in BMDMs, isolated from 4 donor mice, differentiated, and subjected to RNA extraction. Venn diagram of the overlapping up-regulated (D) and down-regulated (E) genes in olaparib- or MNA-treated BMDMs, and biological characterization of the common genes by GO Biological Process analysis (F).

NAD levels are typically tightly maintained and compartmentalized between the nucleus, cytoplasm, and mitochondria (Davila et al., 2018), and NAD is not diffusible through the cell membrane. Therefore, acute substrate accumulation, i.e., free NAD displaced by a catalytic PARP*i*, can be expected to have effects on *de novo* and salvage NAD+ synthesis, such as removal of nicotinamide from the NAD+ salvage pathway via methylation. Given the expected spatial heterogeneity of tumors with areas of active proliferation, areas of necrosis, and yet other areas of fibrosis or immune cell clusters, we used *in situ* mass spectrometry to determine the interplay between olaparib, NAD metabolism, and macrophages. BRCA1f/fp53f/f tumor-bearing mice were treated with olaparib for 10 days, when mice were euthanized and tumors snap frozen, cryosectioned, and thaw mounted onto indium tin oxide (ITO) slides for matrix-assisted laser desorption ionization mass spectrometry imaging (MALDI-MSI) analysis. After establishing a standard curve for olaparib concentrations, we confirmed the presence of olaparib in micromolar concentrations in these tumors (Figures 5A, row 2, and S5A–S5C; Table S1). With regard to NAD metabolites, we detected a decrease in the NAMPT product nicotinamide mononucleotide (Figure 5A, row 3) and a corresponding increase in the levels of methyl-nicotinamide (MNA) in olaparib-treated tumors (Figures 5A, row 5, S5D, and S5E), which was absent after macrophage depletion (Figures S5D and S5E), indicative of reduced NAD salvage and a shift toward methylation of nicotinamide in the setting of NAD abundance resulting from PARP inhibition (Figure 5B). MNA accumulation co-localized to macrophages, here visualized with an F4/80 stain (Figures 5A and S5D). Spearman correlation of the intensity of the MNA signal and the fluorescence signal from the macrophage stain in consecutive tumor sections showed a significant positive correlation for olaparib-treated but not control tumors (Figure S5F). In addition, we observed accumulation of the soluble nicotinate, the conjugate base of nicotinic acid or vitamin B3 required for *de novo* NAD synthesis, in tumor areas with low cellularity (Figure 5A, row 4), while nicotinamide mononucleotide was enriched in the tumor tissue areas with high cellularity (Figures 5A, row 3, S5D, and S5E). *In situ* MS coupled with H&E stain and immunofluorescence demonstrated a large degree of heterogeneity of these tumors with regard to olaparib pharmacokinetics and metabolism, even though they measured less than 20 mm and consisted of a monotonous triple-negative breast cancer (TNBC) population and their TME (Figures 5A and S5D–S5F).

Given MNA’s rise upon olaparib treatment and its ability to diffuse through biomembranes, we examined if MNA could reproduce the macrophage changes observed with PARP inhibition. Although not as potent as olaparib, the NAD derivative MNA was effective at inducing ROS (Figure S5G) and induced the anti-tumor activity of macrophages (Figures 5C and S5H), including in PARP1-null macrophages (Figure S5I). Transcriptionally, about 40% of up- or down-regulated genes was shared between olaparib- and MNA-induced macrophages (Figures 5D and 5E). Up-regulated genes were enriched in inflammatory response- and innate immune system-related pathways (Figure 5F), whereas down-regulated genes were enriched for genes related to RNA processing (Figure 5E; Table S2), consistent with our observation that MNA or olaparib induces differentiation of pro-inflammatory macrophages.

### PARP inhibition of macrophages induces activation of tumor CD8 cells

**Figure 6.**
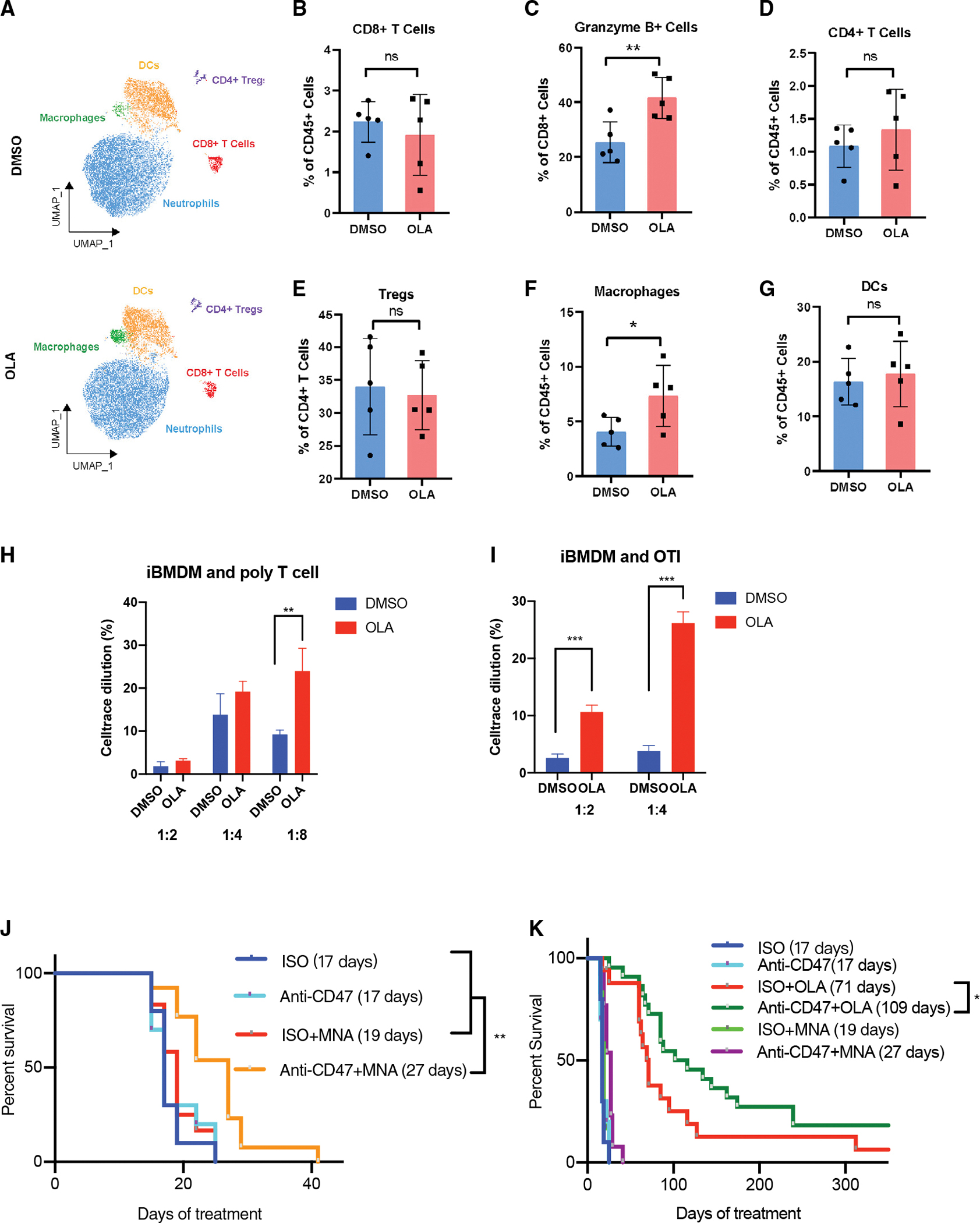
Olaparib-treated macrophages increase macrophage recruitment and enhance CD8 T cell cytotoxicity in the TME (A–G) Autologous, *ex vivo*-induced and olaparib-treated BMDMs were injected intratumorally on days 0 and 7. Mice (5 tumor-bearing mice/arm) were euthanized at day 10, and tumors were subjected to CyTOF. Data are presented as mean ± SD for the respective population. (A) CD45+ cell populations identified by CyTOF. (B–G) Quantitative analysis of subpopulations. (H and I) *Ex vivo*, PARP-inhibitor treatment of macrophages promotes the expansion of CD8^+^ T cells. Immortalized BMDMs (iBMDMs) were co-cultured with T cells from syngeneic wild-type (H; 5 donor mice) or OTI mice (I; 5 donor mice) in the presence or absence of olaparib for 72 h, and CellTrace was added during culture. Cells were stained with anti-CD3 (BV510) and subjected to flow cytometry (5 donor mice for WT or OT1 mice; data are presented as mean ± SD for the respective population). Significance was determined by unpaired Student’s t test. *p < 0.05, **p < 0.01, and ****p < 0.0001. (J and K) Syngeneic implantation of K14 tumors as in Figure 1A. Randomization to isotype control (n = 10) or anti-CD47 (n = 10) antibody as single agents or in combination with olaparib or MNA (50 mg/kg per day orally). Kaplan-Meier curves for MNA treatments with (n = 13) or without CD47 (n = 12) antibodies (J) or for olaparib (n = 22) with or without CD47 (n = 17) antibodies (K). Generation of Kaplan Meier curves and survival analysis with Gehan-Wilcoxon test. Median survival is shown in parentheses.

As effective tumor phagocytosis can also prime a specific T cell response induced by phagocytic macrophages (Tseng et al., 2013), we examined if autologous macrophages pretreated with olaparib could reprogram the adaptive immune system in immunocompetent tumors (Figures 6A–6G and S6A–S6C). *Ex vivo*-differentiated macrophages (Figure 2E) were injected into the tumors of syngeneic, immunocompetent animals. Mice were treated with control or olaparib-treated macrophages only; they did not receive systemic olaparib. Mass cytometry (CyTOF) analysis was conducted 10 days later. Tumor cells and intratumoral leukocyte populations were identified using common lineage-defining markers (Figures 6A, S6A, and S6B). The introduction of olaparib-treated macrophages did not affect tumor cell proliferation, as gauged by Ki67, or PD-L1 expression of tumors (Figure S6C). The absolute number of CD4 and CD8 cells in these highly proliferative TNBCs was low and variable (Figures 6A, 6B, and 6D). We did, however, observe an expansion of the macrophage as well as of the granzyme B-positive cytotoxic T cell population but not regulatory T (Treg) and dendritic cells (Figures 6A–6G and S6A–S6C), consistent with our prior findings (Pantelidou et al., 2019). Consistently, co-culture of immortalized BMDMs (iBMDM) with T cells from syngeneic WT or OTI mice (Curtsinger et al., 1998) showed significant T cell proliferation in olaparib-treated macrophages compared with DMSO controls (Figures 6H, 6I, and S6D).

### Macrophage reprogramming can be harnessed therapeutically using anti-CD47 antibodies *in vivo*

As MNA is bioavailable when taken orally (Tanaka et al., 2015), we examined its effect on tumor growth *in vivo*. We reasoned that weakening the tumor’s defense against macrophage-induced death might enable MNA-treated macrophages to more effectively attack tumor cells. We lowered the tumor’s defense against macrophage-induced cell death and phagocytosis by blocking the “don’t-eat-me” signal, i.e., the immunoglobulin CD47 (Huang et al., 2017; Willingham et al., 2012), which was strongly expressed on tumor cells derived from K14-Cre BRCA1f/fp53f/f tumors (Figure S6E). We found that neither anti-CD47 antibodies nor MNA had single-agent activity in this tumor model. However, the combination of MNA and anti-CD47 antibodies led to a significant, albeit small, survival advantage of 10 days (Figures 6J, S6E, and S6F).

We reasoned that if anti-CD47 antibodies could render the vitamin B3 derivative MNA effective (Figure 6J), it might enhance the efficacy of olaparib to an even greater extent. The combination of anti-CD47 antibodies with olaparib improved median survival from 71 days, similar to what we reported earlier (Pantelidou et al., 2019), to 109 days (Figures 6K and S6F). We quantified granzyme B and interferon-γ levels in CD4 or CD8 cells (Figures S6G–S6K), and while levels were variable, their distribution did not differ between the different treatment modalities. In summary, we found that PARP inhibition in macrophages leads to a reprogramming of TAMs toward an anti-tumor, highly phagocytic phenotype that significantly contributes to the anti-tumor activity of olaparib. This transformation is not dependent on PARP1 or PARP2 but is the result of the accumulation of the PARP substrate NAD+, which allows for increased mitochondrial RET, resulting in ROS production and reprogramming toward an anti-tumor phenotype. The vitamin B3 derivative MNA induces similar changes in macrophages ex vivo and *in vivo*.

## DISCUSSION

PARPis are highly effective to treat homologous recombination deficiency (HRD)-related breast, ovarian, prostate, and pancreatic cancers. While their mechanism of action in tumor cells has been studied in detail, their effects on the TME are largely unknown. We recently found that treatment efficacy with olaparib was reduced when the histologically same tumor was treated in an immunocompromised host (Pantelidou et al., 2019) or when cytotoxic T cells were depleted and that PARP*i*-induced activation of the STING pathway in tumor cells ultimately led to the engagement of cytotoxic T cells (Pantelidou et al., 2019). In that study, we also noted that the largest fraction of immune cells was myeloid cells and specifically macrophages (Pantelidou et al., 2019). Macrophages are a heterogenous population of terminally differentiated monocytes that range in their function from pro-inflammatory and anti-tumor (M1 phenotype) to pro-tumor tissue remodeling (M2 phenotype), induced by IL-4, IL-10, IL-13, and CSF-1. Mehta et al. showed recently that ablation of the latter subpopulation using antibodies against the CSF-1 receptor (CSF-1R) improved the outcomes of olaparib treatments (Mehta et al., 2021). They observed that PARP inhibition led to an expansion of both pro- and anti-tumor macrophages, raising the question of whether PARP inhibition of myeloid cells supports or opposes the anti-cancer effect of PARP*i*s. Through macrophage ablation and reconstitution studies, we found that the net effect of PARP inhibition on TAMs is a strong shift toward an anti-tumor activity (Figures 1 and 2). Strikingly, PARP inhibition can mitigate the M2-inducing effects of IL-4, IL-10, and M-CSF and results in large, highly phagocytic macrophages (Figure 1) with remarkable anti-tumor activity *ex vivo*, suggestive of direct reprogramming of tumor macrophages by PARP inhibition. Olaparib-treated macrophages frequently presented as multi-nucleated giant cells. These can be the result of endoreplication, a process favored by terminally differentiated cells that are highly metabolically active (Edgar and Orr-Weaver, 2001), or of cell fusion, a phenomenon also observed in macrophages and augmented in the presence of IL-4 (Horn and Triantafyllopoulou, 2018). Multi-nucleated giant cells are a recognized feature of phagocytic and highly inflammatory macrophages (McNally and Anderson, 2005). In related studies, the Hottiger group observed that PARP1 inhibition with olaparib greatly enhanced the size and bone-resorbing activity of osteoclasts derived from RAW.264.7 cells (Robaszkiewicz et al., 2016). While in their study of myeloid differentiation toward osteoclasts, the phenotype was strongly dependent on increased transcription of IL-1β, we found in TAMs only a subtle increase in IL-1β transcription and instead a strong switch toward iNOS expression (Figures 3A and 3D). iNOS is a key enzyme in the macrophage inflammatory response that catalyzes the production of cytotoxic nitric oxide (Alderton et al., 2001; Bogdan et al., 2000). Consistent with high PARP*i*-induced expression of iNOS, we found signatures of ROS production, inflammation, and phagocytosis, explaining our observations of an enhanced ability to engulf, kill, and phagocytose tumor cells. This pronounced shift in macrophage function toward an anti-tumor phenotype under the differentiating pressure of M2-inducing cytokines underscores the functional plasticity of TAMs and the ability to pharmacologically modulate their function.

Surprisingly, macrophages were redirected toward a pro-inflammatory, anti-tumor phenotype (Figure 4) if we inhibit the PARP enzymatic activity in WT (PARP1 and PARP2 intact), PARP1-null (PARP2 intact), or PARP2-null (PARP1 intact) cells. Dual PARP KO macrophages could not be established, but as neither PARP1 nor PARP2 were required, we considered that accumulation of the PARP substrate NAD and its derivatives, such as MNA, induce ROS production and macrophage reprogramming. Supporting the key role of ROS, specific inhibitors of ROS generation prevented macrophage reprogramming toward phagocytosis. However, as the transcriptional changes induced by MNA and olaparib only partially overlap (Figure 5), it is possible that the enzymatically inactive or un-PARylated PARP1 or PARP2 additionally contributes to pro-inflammatory gene regulation as reported (Kunze et al., 2019; Meder et al., 2005; Minotti et al., 2015; Robaszkiewicz et al., 2016), aligning the metabolic switch with a transcriptional switch. Other potential mechanisms that enhance transcriptional reprogramming are glycolysis via regulation of hexokinase (Fouquerel et al., 2014), ROS via HIF1α/HIF2α (Izquierdo et al., 2015), or signaling by NAD+ through Sirt1/2 (Schmeisser et al., 2013). The observation that the readily bioavailable MNA could recapitulate the key features of the olaparib-induced reprogramming suggests that MNA is itself an active signaling molecule, as proposed previously (Fukushima et al., 1995; Kilgour et al., 2021; Strom et al., 2018). Our conclusion is that NAD homeostasis and PARylation maintain alternatively activated macrophages and that PARP inhibition allows for a conversion of TAMs to functional anti-tumor macrophages.

Different from chemotherapy, PARP*i*s are not toxic to non-tumor cells with preserved ability to conduct HR and hence are less myelosuppressive. We found that even terminally differentiated macrophages contained a large amount of poly(ADP) ribose (Figure S4A). Consequently, the NAD-competitive olaparib led to a rapid increase in the PARP substrate NAD. Since the oxidized and reduced forms of NAD (NAD+ and NADH, respectively) are co-factors critical for cellular energy hemostasis, accumulation of NAD by PARP inhibition can be expected to promote macrophage energy metabolism. Consistent with this hypothesis, we found increased glycolysis with an increased glycolytic reserve typical of inflammatory macrophages (Figure 3F).

Oxygen consumption at baseline was high in olaparib-treated macrophages, consistent with prior reports that PARP1 ablation increases baseline OCR and glycolysis (Bai et al., 2015; Janko et al., 2021; Modis et al., 2012). But the mitochondrial reserve was markedly reduced, suggesting that oxygen consumption in olaparib-treated macrophages is driven by a process other than mitochondrial ATP production (Figure 3G), i.e., the production of ROS required to kill cancer cells. We found that ROS production was increased in olaparib-treated macrophages (Figure 4) and was rotenone and MitoQ sensitive, consistent with enhanced RET at CI when NAD+ is reduced to NADH to allow for highly efficient ROS formation (Mills et al., 2016; Robb et al., 2018). *In situ* MS provided insight into a high degree of intratumoral metabolic heterogeneity (Figures 5 and S5), as we observed that necrotic areas can serve as reservoirs for olaparib and some metabolites, while MNA maps to areas with high macrophage content (Figure 5).

When a macrophage engages a tumor cell, the decision whether to tolerate or engulf and destroy it is determined by whether or not the macrophage recognizes the tumor cell as “self,” based on its interaction with CD47 and MHCI on tumor cells (Jaiswal et al., 2009; Oldenborg et al., 2000). Here, we find that the anti-tumor activity of macrophages reprogrammed by PARP inhibition can be harnessed through added blockade of the don’t-eat-me signal CD47, a combination that could potentially be studied in clinical trials. As olaparib induces the emergence of cytotoxic T cells (Pantelidou et al., 2019; Figure 6C), and as olaparib in conjunction with anti-PDL1 antibodies is currently under investigation (ClinicalTrials.gov: NCT02849496), a combination of olaparib with the recently discovered bi-specific CD47/PD-L1 antibody (Chen et al., 2021) might also be a consideration. In addition, our findings raise the possibility that enhancing anti-tumor macrophage function with either MNA or olaparib can potentially increase the therapeutic activity of targeted, non-myelosuppressive agents including antibodies or low-dose chemotherapy, all of which are currently being studied in combination with PARP inhibition (Kim et al., 2021).

### Limitations of the study

This work focused on the direct effects of PARP inhibition on TAMs, and we did not examine olaparib’s effects on other immune cells or fibroblasts in the TME. Our data are limited to BC and do not extend to other BRCA1-related cancers such as ovarian, prostate, or pancreatic cancer. Clodronate is taken up by most phagocytic cells and may have effects beyond those associated with the depletion of macrophages. A dual KO of PARP1 and PARP2 would have been desirable to definitively test the necessity for PARP enzymes, but dual germline PARP1/2 KO mice are not viable, and a dual extinction in myeloid cells derived from immortalized bone marrow also did not yield any viable cells in our experimentation. We could only examine paired biopsies from two patients; further translational studies will be possible when materials from ongoing neo-adjuvant or metastatic treatment studies become available.

## STAR★METHODS

Detailed methods are provided in the online version of this paper and include the following:

### RESOURCE AVAILABILITY

#### Lead contact

Further information and requests for resources and reagents should be directed to and will be fulfilled by the lead contact, Gerburg Wulf (gwulf@bidmc.harvard.edu).

#### Materials availability

This study’s unique/stable reagents are available from the lead contact.

#### Data and code availability

The RNA-seq data reported in this publication have been deposited and are available in GEO: GSE210378.

The Mass Spectrometry data have been deposited to the EMBL-EBI MetaboLights database (Haug et al., 2020) (https://doi.org/10.1093/nar/gkz1019, PMID:31691833) with the identifier MTBLS5928. The complete dataset can be accessed here $https://www.ebi.ac.uk/metabolights/MTBLS5928; or on: figshare.com (https://doi.org/10.6084/m9.figshare.20640090; https://doi.org/10.6084/m9.figshare.20639979; https://doi.org/10.6084/m9.figshare.20639895; https://doi.org/10.6084/m9.figshare.20640195. The code developed to analyze Akoya data is available on Github: https://github.com/WulfLab/Macrophage-Quantification.git or https://doi.org/10.5281/zenodo.7071901.

Any additional information required to reanalyze the data reported in this paper is available from the lead contact, Gerburg Wulf (gwulf@bidmc.harvard.edu) upon request.

### EXPERIMENTAL MODEL AND SUBJECT DETAILS

#### Patient-derived tumor sections

The PETREMAC study (clinicaltrials.gov NCT02624973) protocol and clinical trial set-up were approved by the Regional Ethical Committee of the Western health region in Norway (#2015/1493) and The Norwegian Drug Agency (#2015/8463). All patients signed informed consent before inclusion. Patients 1 and 2 were female, 39 and 50 years of age. Archival Formalin-fixed paraffin-embedded tissue sections were processed as described under ‘Immunohistochemistry’.

#### Mouse strains

All animal experiments were conducted in accordance with Institutional Animal Care and Use Committee-approved protocols at Beth Israel Deaconess Medical Center (052-2020). FVB/129P2Ola mice were bred in the BIDMC animal facility. Scid/Beige mice were obtained from Charles River. FVB, Parp1 null, C57BL/6J, OTI and NSG mice were obtained from Jackson Laboratory. Parp2 null mice were kindly provided by Dr. Peter Bai. All of the mice used for tumor implantation were 6–10 weeks old female mice, the mice used for bone marrow extraction were 6–10 weeks old female or male mice.

#### Cell lines

The K14-Cre BRCA1f/fp53f/f (hereafter called K14 cell line) was generated as described (Juvekar et al., 2016). The K14-GFP cell line used for phagocytosis and bioluminescence (BLI) assays was established by transfecting GFP-luciferase into K14 cells. HoxB8 immortalized bone marrow progenitor cells (iBMPCs) were generated as previously described (Wang et al., 2006). Briefly, bone marrow from C57BL6/J mice was transfected with retrovirus containing estradiol-inducible HoxB8 and then maintained in RMPI 1640 complete medium in the presence of 5ng/ml GM-CSF and 1μM β-Estradiol. To generate iBMDM cells, the progenitor cells were washed with PBS and cultured in the RMPI 1640 complete medium with 20 ng/mL MCSF, 20 ng/mL IL-4 and 10 ng/mL and IL10 for 6 days in Petri dishes.

### METHOD DETAILS

#### Cell culture

K14 and K14-GFP Cells were cultured in complete DMEM with 10% Fetal Bovine Serum and 1% penicillin and streptomycin. Bone marrow cells were harvested from mice and cultured in complete RPMI 1640 medium in presence of 20 ng/mL of M-CSF, 20 ng/mL of IL4 and10 ng/ml IL10 to induce bone marrow derived macrophages (BMDMs). Culture medium was exchanged to medium with fresh cytokines at day 3 and cultured for 6 days in total before harvest or subsequent experiments.

#### iBMDM co-culture assay

The iBMDM cells were pulsed with 100 ug/ml OVA protein for overnight or OVA peptide for 1hour. The OT-I cells purified from OT-I TCR transgenic mice were labeled with Celltrace dye (CellTrace™ Violet Cell Proliferation Kit, Thermo fishers) and co-cultured with iBMDM at different ratios. 3 days later, the cells were stained with anti-mouse CD3 BV510 (Biolegend) and analyzed by Cytoflex Flow Cytometer (Beckman).

#### *In vivo* experiments

Pieces from BC tumors generated in K14-Cre Brca1 f/fTrp53f/f female mice (Liu et al., 2007) were transplanted into the mammary fat pad of FVB/129P2Ola or NSG or Scid/Beige recipient females that were at least 6 weeks old. FVB/129P2Ola recipient were generated as described (Pantelidou et al., 2019). For treatment efficacy, imaging and flow cytometry studies, mice were randomized to treatment arm when tumors reached 5+/−1 mm in diameter and treatments continued until tumors reached 20 mm in the largest dimension, the pre-defined endpoint at which mice were euthanized. DMSO-reconstituted olaparib was diluted in PBS (Corning) immediately before intraperitoneal injection and administered at 50 mg/kg daily. Anti-CD47 and IgG2b isotype control antibodies were dissolved in PBS and administered intraperitoneally at 0.4 mg/dose twice per week in the first week and subsequently one per week. Tumors were measured every 2 or 3 days using electronic calipers, and tumor volumes were calculated by using the ellipsoid formula for volume (L × W × W/2).

#### Immunoblotting

Cells were lysed in RIPA buffer supplemented with protease inhibitors (Roche), quantitated for protein using the Bradford Protein Assay Kit (Pierce), and equal amounts of protein were resolved by SDS-PAGE (Bio-Rad). Subsequent procedures were performed following Li-COR system protocol.

#### Bioluminescence assay (BLI assay)

BMDM cells were harvested at day 6 and seeded in 96 well plates, different numbers of cells were seeded as indicated. After 4 h, 5000 K14-GFP cells were added to each well. Cells were co-cultured in the presence of cytokines for another 24 h. Then removed the medium, washed with PBS once and added fresh medium with D-luciferin (150μg/ml) and incubated at 37°C for 10min, read luminescence immediately.

#### Real-time PCR

Total RNA was isolated from BMDM using Trizol (Invitrogen). cDNA synthesis was performed using iScript cDNA synthesis kit (Bio-Rad). cDNA samples were used for quantitative real-time PCR analysis with iQ SYBR Green supermix and PCR was performed on a 7500 HT Fast Real-Time PCR System (Applied Biosystems). The SYBR primer pair sequences were available in Table S3. Fold changes in expression were calculated by the Delta Delta Ct method using mouse 18S as an endogenous control for mRNA expression. All fold changes are expressed normalized to the untreated control.

#### Transcriptomics analysis

Total RNA was isolated using Trizol (Invitrogen). RNA-seq libraries were constructed using Illumina Stranded mRNA Prep kit as described in the manual and subjected to 150-bp single end sequencing on HiSeq 2500. STAR aligner (version 2.5.2b) was used to align reads to the mouse genome (GRCm38.p5) using annotation from Ensembl release 99 with reads-per-gene count enabled as described in the manual. The DEseq2 package was for differential expression analysis using R statistical software (version 3.6.0). For expression level visualizations, we used regularized log transformation of the count data, as described in the package vignette. To test whether there were any sets of related genes in the list of differentially expressed genes we used the gage-package in combination with gene sets from MSigDB. Correction for multiple testing was performed using false discovery rate with the Benjamini-Hochberg method.

#### Mass spectrometry for metabolites in BMDM

BMDM culture medium was aspirated thoroughly, place the dishes on dry ice, and add 4mL of −80°C prechilled 80% methanol. Place dishes in −80°C freezer for 20min. Scrape cells on dry ice and collect all the mixture to 15mL tubes. Centrifuge the mixture at 13000 rpm for 10min, collect supernatant to a new tube, and resuspend the pellet with 1mL of −80°C prechilled 80% methanol. Centrifuge and collect the supernatant and pool together with former extraction. Dry the extraction by using SpeedVac in room temperature. Dissolve the dried metabolites in 20μL HPLC grade water and run the samples on an AB/SCIEX 550 QTRAP Mass Spectrometry.

#### *In situ* mass spectrometry

##### Mass spectrometry imaging tissue preparation

K14-Cre BRCA1f/fp53f/f tumor-bearing mice were treated with olaparib for 10 days. The orthotopic tumors were extracted and snap frozen in liquid nitrogen, cryosectioned at 10 μm thickness, and thaw-mounted onto indium tin oxide (ITO) slides for MALDI-MSI analysis. Serial sections were stained using hematoxylin and eosin (H&E) and imaged using a 10× objective (Zeiss Observer Z.1, Oberkochen, Germany) to produce a high-resolution whole tissue image. A tissue mimetic made of homogenized control mouse brain tissue was spiked with olaparib concentrations ranging from 1.0–20 μM. The spiked homogenates were dispensed into a six-channel tissue microarray array (TMA) mold composed of 40% gelatin and frozen. The olaparib tissue mimetic model was processed similarly to the tissue sections.

##### MALDI MSI matrix and instrumental parameters

Three separate MALDI matrices were prepared to capture the spatial distribution of olaparib, 1-MNA, nicotinamide mononucleotide, and nicotinic acid. Each matrix solution was applied to a serial section of the tissues. Olaparib was imaged using 2,5-dihydroxybenzoic acid (160 mg/mL) matrix solution dissolved in 70:30 methanol: 0.1% TFA with 1% DMSO. 1-MNA and nicotinic acid were imaged using α-cyano-4-hydroxycinnamic acid (5 mg/mL) matrix in 70:30 methanol: water with 0.1% TFA. Nicotinamide mononucleotide was imaged using 1,5-diaminonaphthalene hydrochloride (4.3 mg/mL) matrix in 4.5/5/0.5 HPLC grade water/ethanol/1 M HCl (v/v/v). All matrices were applied using a TM-sprayer (HTX imaging, Carrboro, NC) and instrumental parameters are found in Table S1. Mass spectrometry imaging data acquisition was performed using the timsTOF fleX mass spectrometer (Bruker Daltonics, Billerica, MA). Data acquisition methods were optimized made for each analyte by direct infusion of relevant standards into the ESI source to optimize ion transfer funnels, quadrupole, collision cell, and focus pre-TOF parameters which were transferred a MALDI data acquisition method. The mass range was calibrated for each run using the Agilent tune mix solution (Agilent Technologies, Santa Clara, CA).

##### MALDI MRM MSI

The instrument was operated in positive ion mode for olaparib imaging and set to multiple reaction monitoring (MRM) covering the *m/z* 100–2000 range. Collision induced dissociation energy settings for the olaparib precursor ion was set to 35 eV with a 3 *m/z* isolation window. The precursor to product ion transition 435.183→281.072 was used to monitor olaparib, corresponding to [C_24_H_23_FN_4_O_3_+H]^+^ and [C_16_H_9_FN_2_O_2_+H]^+^ (Figure S5A). Olaparib was imaged with a 10,000 Hz laser repetition rate and 1,000 laser shots per 100 μm pixel size. A linear regression (R^2^ = 0.989) was calculated from the olaparib calibration curve correlating the ion intensities to spiked olaparib concentrations between 0 to 5 μM range (Figures S5B and S5C). A limit of detection (LOD) of 0.9 μM (S/N ratio of >3) and limit of quantification (LOQ) of 2.9 μM (S/N ratio of >10) were calculated.

##### MALDI MSI

Nicotinic acid and 1-MNA ion distributions were imaged from the same tissue section using CHCA matrix in positive ion mode. The MALDI MSI data was acquired between *m/z* 50–1050 with a laser repetition rate of 2,000 Hz and 1,000 laser shots per 100 μm pixel size. Nicotinamide mononucleotide was imaged using the timsTOF fleX mass spectrometer in negative ion mode between *m/z* 50–1050 (laser repetition rate was set to 2,000 Hz with 1,000 laser shots per 100 μm pixel size). A 500 nL spot of each standard was applied onto the ITO slide to confirm the *m/z* ion selection for the MSI imaging with a mass error <10 ppm for each analyte. MSI data was visualized using the SCiLS Lab software (version 2021a premium, Bruker Daltonics, Billerica, MA).

#### Seahorse assay

BMDM were seeded at 1 × 10^5^ cells/well in RPMI-1640 in a Seahorse XF24 Cell Culture plate. The Seahorse XF Glycolysis Stress Test Kit and Seahorse XF Cell Mito Stress Test Kit (Seahorse Bioscience) were used to detect the extracellular acidification rate (ECAR) and cellular oxygen consumption rate (OCR). The plate was detected following the instructions. For ECAR detection, glucose (10 mM), oligomycin (1 μM), and 2-DG (50 mM) were sequentially injected into each well at indicated time points. For OCR detection, oligomycin (1.5 μM), FCCP (0.25 μM) and rotenone/antimycin A (0.5 μM) were sequentially injected.

#### NAD^+^ detection

BMDM were harvested and lysed in assay buffer. The supernatant was collected after 10min of centrifuge at speed of 13,000 rpm for 10min. Add supernatant to a 96 well plate and reagents in the kit. NAD^+^ content was measured at OD450 nm according to manufacturer’s instruction. The NAD^+^ content was normalized to protein concentration of each sample.

#### Immunohistochemistry and *H&E*

##### H&E staining

Mice were euthanized when the tumor reached end point. Tumors were dissected and fixed in SafeFix and embedded in paraffin. 5 mm sections were prepared and stained with hematoxylin and eosin (H&E).

##### Immunohistochemistry

After xylene de-paraffinization and rehydration through graded ethanol antigen retrieval was performed for 20 min at 100°C with 0.1% sodium citrate buffer (pH 6.0). Following quenching of endogenous peroxidase activity with 3% H_2_O_2_ and blocking of non-specific binding with 5% bovine serum albumin buffer, sections were incubated overnight at 40°C with the appropriate primary antibodies followed by incubation with 1:200 biotinylated secondary antibodies for 30 min and 1:500 streptavidin-HRP for 30 min. Bound peroxidase was visualized by 1–10 min incubation in a 3, 30-diaminobenzidine (DAB) solution (Vector Laboratories, SK-4100). For double stain IHC, the procedures were performed according to the manufacturer’s instruction (Abcam). Slides were photographed on an upright light/fluorescent Imager A2 microscope with AxioVision Release 4.5 software (Zeiss, Germany). Antibodies used in IHC were iNOS (Thermo PA1-036), Mac2 (Biolegend, 125403), γH2AX (CST, 9718T).

##### Immunohistochemical evaluation (by Akoya Opal IHC kit)

We performed the OpalTM 7-color cyclic immunofluorescence (IF) assay (Akoya Biosciences, Marlborough, MA). We stained for six markers and nuclei: 1) iNOS (1:200 dilution; catalog number: PA1-036; ThermoFisher), 2) F4/80 (1:200 dilution; catalog number: ab111101; Abcam), 3) IFN gamma (1:100 dilution; catalog number: 513202; Biolegend); 4) Granzyme B (1:500; catalog number: ab255598; Abcam), 5) CD4 (1:750; catalog number: ab183685; Abcam), 6) CD8 (1:100; catalog number: ab217344; Abcam), 7) nucleus with 4,6-diamidino-2-phenylindole (DAPI; Akoya Biosciences). The pairings of each of the 6 markers with an OpalTM fluorophore and the order of antibody staining were optimized as follows: cycle 1 was CD8 paired with OpalTM fluorophore 620, cycle 2 was Granzyme B with 570, cycle 3 was IFN gamma with 520, cycle 4 was iNOS with 480, cycle 5 was CD4 with 690, and cycle 6 was F4/80 with 780. OpalTM 780 is an antibody-based reaction that requires the use of tyramide signal amplification-digoxigenin (TSA-DIG) for signal amplification, as such, F4/80 paired with OpalTM 780 had to be stained last.

The sections were baked at 65°C for three hours before placing the slides into the Bond RX fully automated research stainer (Leica Biosystems, Deer Park IL) for dewaxing and the OpalTM assay. The OpalTM assay began with a 40 mins of heat-induced epitope retrieval step at 100°C using the Bond epitope retrieval solution 2 (pH 9), followed by the five cycles of blocking (5 mins), primary antibody incubation (30 mins), incubation with OpalTM polymer horseradish peroxidase (HRP) reagent (10 mins), signal amplification with the marker’s paired OpalTM fluorophore (10 mins), and antibody stripping using Bond epitope retrieval solution 1 (pH 6) at 95°C for 20 mins. The sixth cycle to stain for F4/80 was slightly modified: blocking, F4/80 antibody incubation, HRP incubation, using the OpalTM TSA-DIG for signal amplification (10 mins), stripping of TSA-DIG, and incubation with OpalTM 780 for signal generation (60 mins). The slides were last stained for DAPI (5 mins). There were three to four washes in between each step. Slides were mounted with ProLong™ gold antifade mountant (Thermo Fisher Scientific, Waltham, MA). IF images were visualized using Phenochart (Akoya Bioscience). IF-stained slides were digitized at 40× by PhenoImager HT (formerly Vectra Polaris, Akoya Biosciences, Marlborough, MA). The images were analyzed using inForm ® (version 2.5) by building two algorithms to detect and quantify cells of interest. The first algorithm quantified CD8+ and CD4+ cells, in conjunction with GzB + or IFN+. The second algorithm quantified iNos + cells, with and without F480+.

#### Immunofluorescence assay

Frozen tumor slides were fixed in cold acetone for 20 min in −20°C. Dry the slides in room temperature for 5min and rehydrate in PBS for 10min. Block the section with horse serum at room temperature for 30min, following with primary antibody incubation overnight in 4°C. Wash the section and incubate with fluorescent secondary antibody for 1h at room temperature. Wash the section with PBST and mount the slide with gold antifade mounting medium. Images are taken by using a Keyence microscope or a confocal microscope (Zeiss 510). Antibodies used in IF: F4/80 (ab6640)

#### Flow cytometry

##### For phagocytosis assay

BMDM cells were cocultured with K14-GFP cell for 4hours. Cells were harvested and stained with F4/80, cells were then subject to flow cytometry.

##### For ROS detection

BMDMs were washed by PBS before scraping and collecting to FACS tubes. Cells were centrifuged at 1500rpm for 5min and resuspended in the buffer containing DCFDA (Abcam). Cells were incubated in 37°C for 30min. Tubes were covered by tinfoil and placed in the ice. ROS were analyzed using a CytoFLEX flow cytometer.

##### For cell immunophenotype analysis

BMDM cells were harvested and resuspended in 1% FBS PBS buffer. Spined down the cell at 1500rpm, removed the supernatant. Cells were resuspended with CD16/CD32 dilution and incubated at 4°C for 10 min. Cells were centrifuged at 1500rpm for 5min, and resuspended in CD206 (Biolegend, 141709), CD86 (Biolegend, 105013) and F4/80 (Biolegend, 123121) antibody dilutions and incubate for 30min at 4°C. After incubation, the cells were centrifuged and washed with 1% FBS PBS. Cells were analyzed by flow cytometry with a Cytoflex(Beckman).

#### Mass cytometry

##### Antibody staining for mass cytometry

Except where indicated sample staining and acquisition were carried out at room temperature. Mouse tumor tissues were dissociated into single-cell suspension using the Tumor Dissociation Kit (Miltenyi Biotec) and the gentleMACS™ Octo Dissociator following manufacturer’s instructions. Cells were stained with Cisplatin-195Pt at a final concentration of 1 μM for 5 min. After viability staining, cells were incubated with Fc-Receptor blocking solution. Fifteen minutes later, the surface staining antibody cocktail was added to each cell suspensions and incubated for 30 min without washing out the Fc blocking. The cells were then washed with Maxpar Cell Staining Buffer (CSB) (Fluidigm) for a total of two wash. Then cells were incubated with Nuclear Antigen Staining Buffer (Fluidigm) with gentle vortex for 30 min. After two washes with Nuclear Antigen Staining Perm (Fluidigm), cells were stained with secreted and nuclear antigen antibody cocktail for 30 min. Following the staining, cells were washed twice with Nuclear Antigen Staining Perm and fixed by freshly made 1.6% paraformaldehyde for 10 min. Afterwards, cells were incubated with Cell-ID Intercalator-Ir (Fluidigm) overnight at 4°C. Cells were then washed in CSB buffer and with subsequent washes in Cell Acquisition Solution (CAS) (Fluidigm) to remove buffer salts and cell debris for total of two washes. Immediately prior to sample acquisition, cells were resuspended at 5~6 × 10^5^ cells per mL in CAS containing EQ™ Four Element Calibration Beads (1:5) (Fluidigm) and filtered through a 40 μm cell strainer.

##### Mass cytometry acquisition setting and data analysis

For quality control, the acquisition event rate was maintained under 500 events/s, and the EQ™ beads were confirmed to have clustered events >10,000 and median Eu151 and Eu153 intensity were over 1000 to ensure appropriate mass sensitivity. Original data acquired by CyTOF were randomized and normalized using the FSC processing function of the CyTOF software. The Gaussian Parameters were applied to gating the FSC processed files using FlowJo. Standard gating strategies were used for single cell analysis with multiple markers. The populations of interest were gated to visualize the high-dimensional data and identify clusters of cells with a similar expression of cell surface markers in CyTOF. The UMAP algorithm was applied to data from a certain number of randomly selected cells from each sample. Clustering analysis was performed using the PhenoGraph implementation in the FlowJo plugins. The resulting PhenoGraph clusters were projected onto the UMAP. Cluster Explorer plugin in FlowJo was performed to define the cell clusters by typical marker expression. For hierarchical clustering, the distances between clusters were computed using the Euclidean measurement method. Dendrograms were generated using average linkage. A normalized heatmap for each marker within all generated clusters was displayed. For the pairwise correlation heatmap, the correlations between all pairs of parameters were calculated using the Spearman correlation and displayed in a heatmap. All cytometry data can be made available upon request.

### QUANTIFICATION AND STATISTICAL ANALYSIS

Statistical analyses were performed using GraphPad Prism v8. For the *in vitro* experiments comparisons between two groups were calculated using an unpaired t-test, comparisons for multiple groups were calculated using one-way or two-way ANOVA. Data are reported as mean ± SD. For the *in vivo* experiments, growth curves and Kaplan Meier curves of survival were graphed, survival between cohorts was compared using log-rank or Wilcoxon tests. *p < 0.05, **p < 0.01, ***p < 0.001. For *in vitro* experiments, n = number of separate experiments. For *in vivo* work, n = number of individual animals. Statistical details can be found in the figure legends.

### ADDITIONAL RESOURCES

The clinical registry number of the PETREMAC study: clinicaltrials.gov NCT02624973.

## Supplementary Material

1

## Figures and Tables

**KEY RESOURCES TABLE:** 

REAGENT or RESOURCE	SOURCE	IDENTIFIER

Antibodies		

Rabbit anti-iNOS	Cell signaling Technology	Cat#13120; RRID: AB_2687529
Rabbit anti-iNOS	Thermo Fisher Scientific	Cat#PA1-036; RRID: AB_325773
Rabbit anti-PARP1	Cell signaling Technology	Cat#9532; RRID: AB_659884
Mouse anti-PAR	Trevigen	Cat#4335-MC-100; RRID:AB_2572318
Mouse anti-Actin	Cell signaling Technology	Cat#3700; RRID:AB_2242334
Mouse anti-CD47	Novusbio	Cat#NBP2-31106
*InVivo*MAb anti-mouse CD47 (IAP)	Bioxcell	Cat#BE0270; RRID:AB_2687793
*InVivo*MAb rat IgG2a isotype control	Bioxcell	Cat#BE0089; RRID:AB_1107769
Rat anti-MAC2	Biolegend	Cat#125403; RRID:AB_1236484
Rabbit anti-Phospho-Histone H2A.X (Ser139)	Cell signaling Technology	Cat#9718; RRID:AB_2118009
Rabbit anti-F4/80	Abcam	Cat#ab111101; RRID:AB_10859466
Rat anti-F4/80	Abcam	Cat#ab6640; RRID:AB_1140040
Rat anti-F4/80	Biolegend	Cat#123121; RRID:AB_893492
Mouse anti-IFNγ	Biolegend	Cat#513202; RRID:AB_1089144
Rabbit anti-GranzymeB	Abcam	Cat#ab255598; RRID:AB_2860567
Rabbit anti-CD4	Abcam	Cat#ab183685; RRID:AB_2686917
Rabbit anti-CD8	Abcam	Cat#ab217344; RRID:AB_2890649
Rat anti-CD206	Biolegend	Cat#141709; RRID:AB_10933252
Rabbit anti-CD206	Proteintech	Cat# 18704-1-AP; RRID:AB_10597232
Rat anti-CD86	Biolegend	Cat#105013; RRID:AB_439782
Rabbit anti-Arginase	Cell signaling Technology	Cat#93668; RRID:AB_2800207
Anti- CD25 - 151Eu	Fluidigm	Cat# 3151007B; RRID:AB_2827880
Anti-Mouse CD3e (145-2C11)-152Sm	Fluidigm	Cat# 3152004; RRID:AB_2687836
Anti-CD45R/B220 (RA3-6B2)-176Yb	Fluidigm	Cat# 3176002B; RRID:AB_2895123
Anti-Mouse NK1.1 (PK136)-170Er	Fluidigm	Cat# 3170002B; RRID:AB_2885023
Anti-Mouse CD11c (N418)-142Nd	Fluidigm	Cat# 3142003B; RRID:AB_2814737
Anti-Mouse CD11b (M1/70)-148Nd	Fluidigm	Cat# 3148003B; RRID:AB_2814738
Anti-Mouse CD8a (53-6.7)-146Nd	Fluidigm	Cat# 3146003B; RRID:AB_2687833
Anti-Mouse CD4 (RM4-5)-145Nd	Fluidigm	Cat# 3145002B; RRID:AB_2687832
Anti-Human CD45RA (HI100)-155Gd	Fluidigm	Cat# 3155011B; RRID:AB_2810246
Anti-Ly-6G/C (Gr-1)-141Pr	Fluidigm	Cat# 3141005B; Clone: RB6-8C5
Anti-Mouse CD274/PD-L1 (10F.9G2)-153Eu	Fluidigm	Cat# 3153016; RRID:AB_2687837
Anti-CD152 (CTLA-4)-154Sm	Fluidigm	Cat# 3154008B; clone: UC10-4B9
Anti-Mouse Foxp3 (FJK-16s)-158Gd	Fluidigm	Cat# 3158003A; RRID:AB_2814740
Anti-Mouse CD279/PD-1 (29F.1A12)-159Tb	Fluidigm	Cat# 3159024; RRID:AB_2687839
Anti-Human/Mouse Granzyme B-173Yb	Fluidigm	Cat# 3173006B; RRID:AB_2811095
Anti-Mouse MHC Class I (28-14-8)-144Nd	Fluidigm	Cat# 3144016B; RRID:AB_2687831
Anti-CD326 (EpCAM)-166Er	Fluidigm	Cat# 3166014B; clone: G8.8
Anti-IFNg-165Ho	Fluidigm	Cat# 3165003B; clone: XMG1.2
Anti-Ki-67 (B56)-162Dy	Fluidigm	Cat# 3162012B; RRID:AB_2888928

Biological samples		

Patient-derived tumor sections	Hans P. Eikesdal Lab	N/A

Chemicals, peptides, and recombinant proteins		

Olaparib	MCE	Cat#HY-10162
H2DCFDA (H2-DCF, DCF)	ThermoFisher	Cat#D399
N-Methylnicotinamide (MNA)	FisherScientific	Cat#M037425G
Mitoquinone (MitoQ10) mesylate	Selleck	Cat#S8978
Rotenone	Agilent	Cat#103015-100
Trolox	MCE	Cat#HY-101445
MitoSOX	ThermoFisher	Cat#M36008
β-Estradiol	Sigma-Aldrich	Cat#E8875
Clodronate Liposomes & Control Liposomes (PBS)	Liposoma	Cat#CP-050-050
Recombinant murine IL-4	Peprotech	Cat#214-14
Recombinant murine M-CSF	Peprotech	Cat#315-02
Recombinant mouse IL-10	Prospec Bio	Cat#CYT-497
Recombinant mouse GM-CSF	Peprotech	Cat#315-03
IVISbrite D-Luciferin Potassium Salt Bioluminescent Substrate (1g) (XenoLight)	PerkinElmer	Cat#122799

Critical commercial assays		

Qtracker™ 625 Cell Labeling Kit	ThermoFisher	Cat#A10198
NAD/NADH Quantitation Colorimetric Kit	Biovision	Cat#K337
CellTrace™ Violet Cell Proliferation Kit	ThermoFisher	Cat#C34557
Double stain IHC kit	Abcam	Cat#ab183285
Opal 7-Color IHC Kit	Akoya	Cat# NEL811001KT
Seahorse XF Glycolysis Stress Test Kit	Seahorse Bioscience	Cat#103020-100
Seahorse XF Cell Mito Stress Test Kit	Seahorse Bioscience	Cat#103015-100

Deposited data		

RNA seq data	This paper	GEO: GSE210378
Mass Spectrometry data	This paper	https://doi.org/10.6084/m9.figshare.20640090 https://doi.org/10.6084/m9.figshare.20639979 https://doi.org/10.6084/m9.figshare.20639895 https://doi.org/10.6084/m9.figshare.20640195 EMBL-EBI MetaboLights database https://doi.org/10.1093/nar/gkz1019, PMID:31691833 with the identifier MTBLS5928
Code for Akoya data analysis	This paper	https://github.com/WulfLab/MacrophagM-Quantification.git or https://doi.org/10.5281/zenodo.7071901

Experimental models: Cell lines		

K14-GFP	This paper	N/A
iBMPC	This paper	N/A

Experimental models: Organisms/strains		

Mouse: FVB/129P2Ola	This paper	N/A
Mouse: NOD.Cg-*Prkdc^scid^ Il2rg^tm1Wjl^*/SzJ (NSG)	Jackson Laboratory	RRID: IMSR_JAX:005557
Mouse: CB17.Cg-PrkdcscidLystbg-J/Crl (Scid/Beige)	Charles River	RRID: IMSR_CRL:250
Mouse: 129S-Parp1tm1Zqw/J (Parp1 null)	Jackson Laboratory	RRID: IMSR_JAX:002779
Mouse: Parp2 knockout	Peter Bai Lab	N/A
Mouse: C57BL6/J	Jackson Laboratory	RRID: IMSR_JAX:000664
Mouse: C57BL/6-Tg(TcraTcrb)1100Mjb/J (OTI)	Jackson Laboratory	RRID: IMSR_JAX:003831
Mouse: FVB/NJ (FVB)	Jackson Laboratory	RRID: IMSR_JAX:001800

Oligonucleotides		

qPCR primers for Figures 3 and S4, see Table S3	This paper	N/A

Software and algorithms		

Prism 8	Graphpad	RRID: SCR_002798
Biorender	biorender.com	RRID: SCR_018361
ImageJ	https://imagej.net/	RRID:SCR_003070
FlowJo	FlowJo	https://www.flowjo.com/solutions/flowjo/downloads
inForm® (version 2.5)	Akoyabio	https://www.akoyabio.com/phenoimager/software/inform-tissue-finder/
CyTOF Helios	Fluidigm	https://go.fluidigm.com/cytof

## Highlights

- PARP inhibition reprograms metabolism and transcription in macrophages
- Accumulation of NAD increases reactive oxygen species via reverse electron transport
- High ROS production and phagocytic capacity result in anti-tumor function
- The combination of olaparib with CD47 antibodies improves outcomes in a mouse model
