# Supplementary material for: PARP-inhibition reprograms macrophages toward an anti-tumor phenotype
Source: Cell Rep. Author manuscript; Available in PMC 2022 Dec 7. (PMC9727835; doi:10.1016/j.celrep.2022.111462)
Supplement: 1 [file NIHMS1842064-supplement-1.pdf]

Cell Reports, Volume *41*

Supplemental information

PARP-inhibition reprograms macrophages toward an anti-tumor phenotype

Lin Wang, Dan Wang, Olmo Sonzogni, Shizhong Ke, Qi Wang, Abhishek Thavamani, Felipe Batalini, Sylwia A. Stopka, Michael S. Regan, Steven Vandal, Shengya Tian, Jocelin Pinto, Andrew M. Cyr, Vanessa C. Bret-Mounet, Gerard Baquer, Hans P. Eikesdal, Min Yuan, John M. Asara, Yujing J. Heng, Peter Bai, Nathalie Y.R. Agar, and Gerburg M. Wulf

A

	Breast Cancer Cell lines	Ovarian Cancer Cell Lines
Number of values	43	29
Minimum	0.8998	0.8770
25% Percentile	0.9305	0.9273
Median	0.9463	0.9538
75% Percentile	0.9562	0.9706
Maximum	0.9799	0.9911
Range	0.08011	0.1141

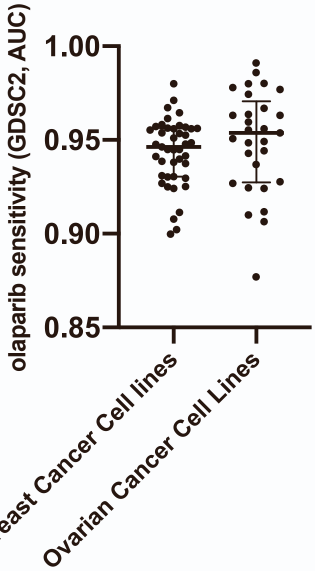

B

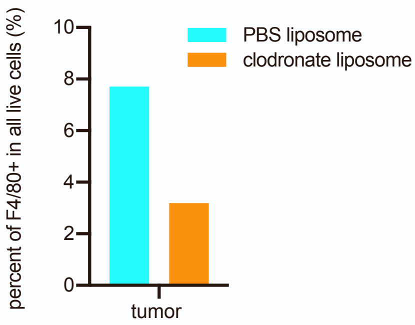

C

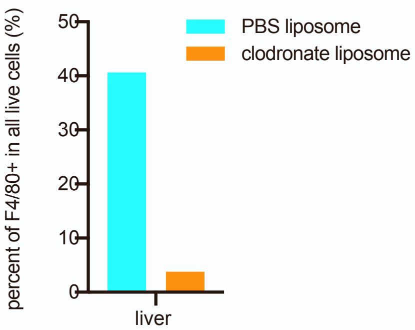

D

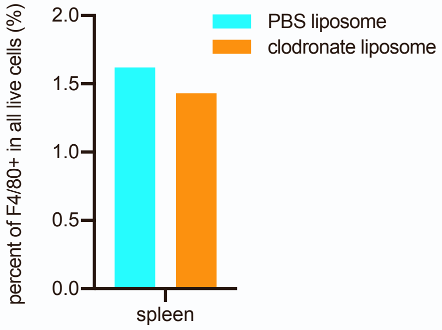

E

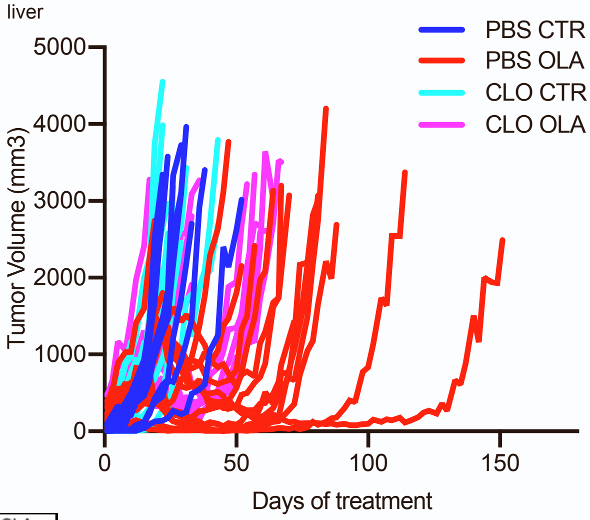

F

	PBS CTR	PBS OLA	CLO CTR	CLO OLA
Mean	45.083454	201.7817557	113.800022	249.8765725
Median	41.68796	195.3308	114.788935	210.8605
significance (p value)	PBS OLA and CLO OLA (n.s.)			

G

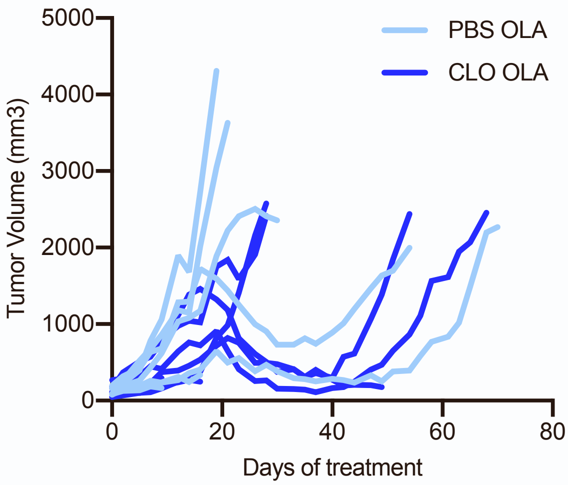

H

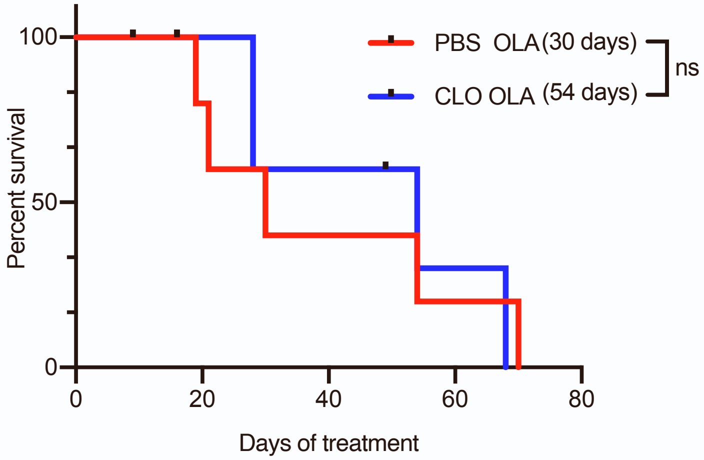

Figure S1. Olaparib augments anti-tumor macrophage activity. Related to Figure 1

(A) Olaparib sensitivity assay of breast cancer and ovarian cancer cell lines, data downloaded from Genomics of Drug Sensitivity in Cancer database (GDSC21017).

(B-D) Validation of clodronate to deplete macrophages. Tumor-bearing mice (n=2) were randomized to clodronate liposome or PBS control liposomes for 1 week according to scheme in Fig. 1A. Tumors, livers and spleens were harvested and dissociated into single cells. Cells were stained with F4/80 and live/dead cell dye (Zombie aqua) and subjected to flow cytometry. F4/80 positive cells were gated and analyzed in tumors (B), livers (C) and spleens (D).

(E-F) Tumor growth curves of mice treated with olaparib or vehicle alone or in combination with clodronate liposome or PBS control, corresponding to Fig1B (E) and tumor volumes at the time of randomization were equally distributed between cohorts (F) Tumor volumes at study entry were compared using an unpaired T-test.

(G-H) K14 tumors were implanted in SCID/beige mice (congenic immunodeficient CB17.Cg-$\textit{Prkdc}^{scid}\textit{Lyst}^{bg\text{-}J}$/Crl). Mice were treated with olaparib in combination with clodronate liposome (n=5) or PBS liposome control (n=5) when the tumor reached 4-6 mm in diameter. Tumor growth curves (G) Study end point was 20 mm in longest dimension of tumor, Kaplan-Meier curves of survival on treatment until endpoint in (H).

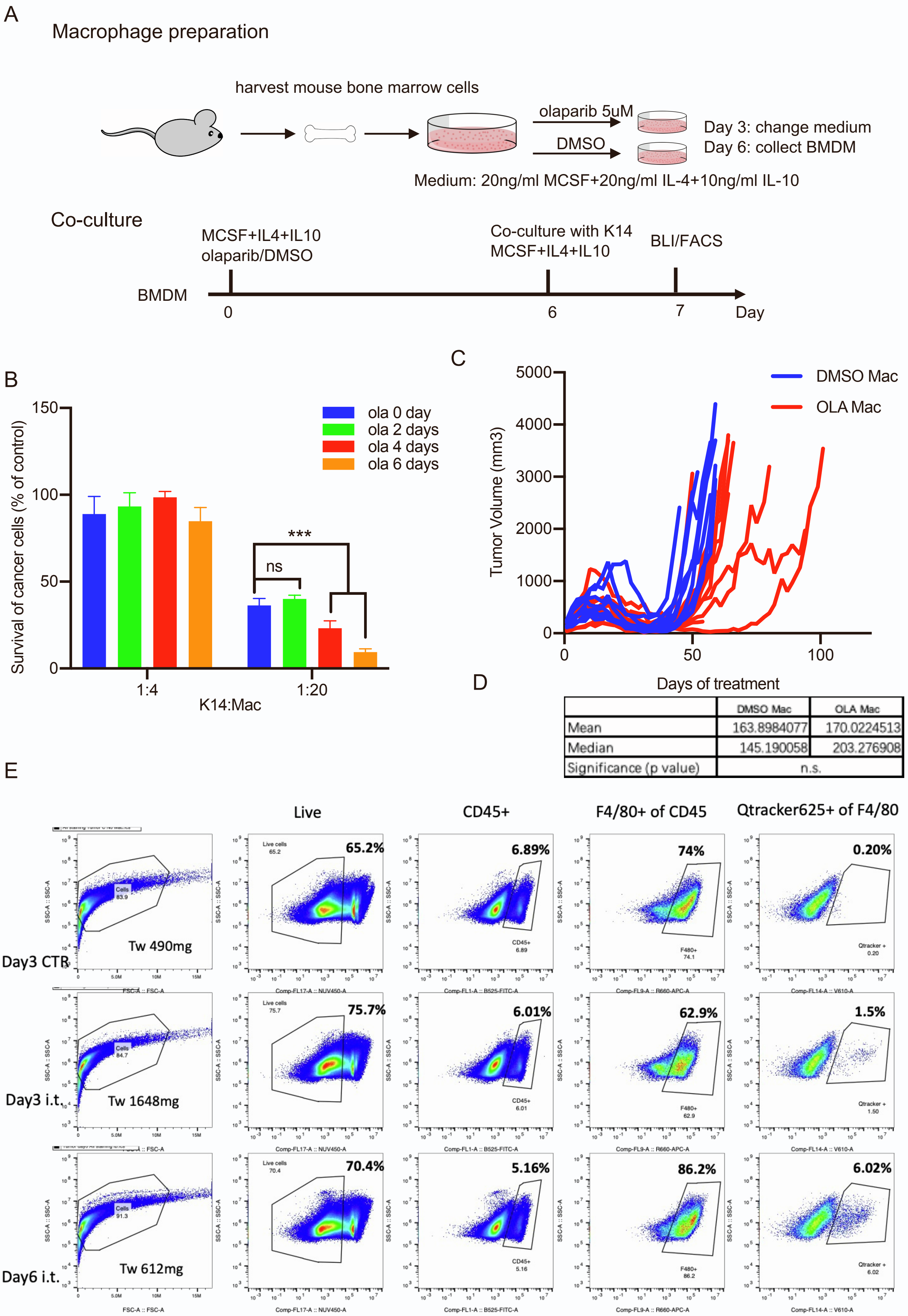

	DMSO Mac	OLA Mac
Mean	163.8984077	170.0224513
Median	145.190058	203.276908
Significance (p value)	n.s.	

Figure S2. Olaparib directly reprograms alternatively activated macrophages towards an anti-tumor activity. Related to Figure 2

(A) Schema of *ex vivo* culture procedures of BMDM and co-culture procedure with K14-GFP cancer cells.

(B) Bone marrow cells were isolated, seeded and cultured in the presence of cytokines as indicated for 6 days to yield BMDMs and treated as indicated. Cells were co-cultured with K14-GFP cancer cells at ratios as indicated, phagocytosis of BMDM was analyzed via BLI assay. Data represent mean +/- SD of 6 replicate cultures derived from BM from 6 donor mice. Significance determined using two-way ANOVA.

(C-D) Corresponding to Fig. 2 E,F. K14 tumors were implanted in NSG (NOD.*Cg-Prkdc*^{scid}*Il2rg*^{tm1Wjl}/SzJ) mice (10 tumor-bearing mice/arm). Macrophages treated with olaparib and DMSO *ex vivo* were intratumorally injected when the tumor reached 4-6 mm in dimension. Tumor growth curves (C) and tumor volumes at the time of randomization (D).

(E) Determination of viability of intratumorally injected macrophages. NSG mice (n=3) were implanted with K14 tumors, and these tumors were allowed to reach a diameter of 4-6 mm. Bone marrow-derived macrophages were generated as described in A, from a total of 6 donor mice, in the presence of cytokines but absence of Olaparib. After 6 days, they were labeled with Q-tracker, a total of 1x10^6 cells were injected intratumorally. Mice were euthanized 3 or 6 days later, tumor weight was measured, tumors were dissociated and cell content analyzed via flow cytometry. Gating strategy for flow cytometry: Cells were gated in the forward versus side scatter dot plot first, then dead cells were excluded from analysis by use of Zombie Aqua, next CD45 and F480 dual-positive cells were selected and finally the Q-tracker positive population, representing injected macrophages, quantified.

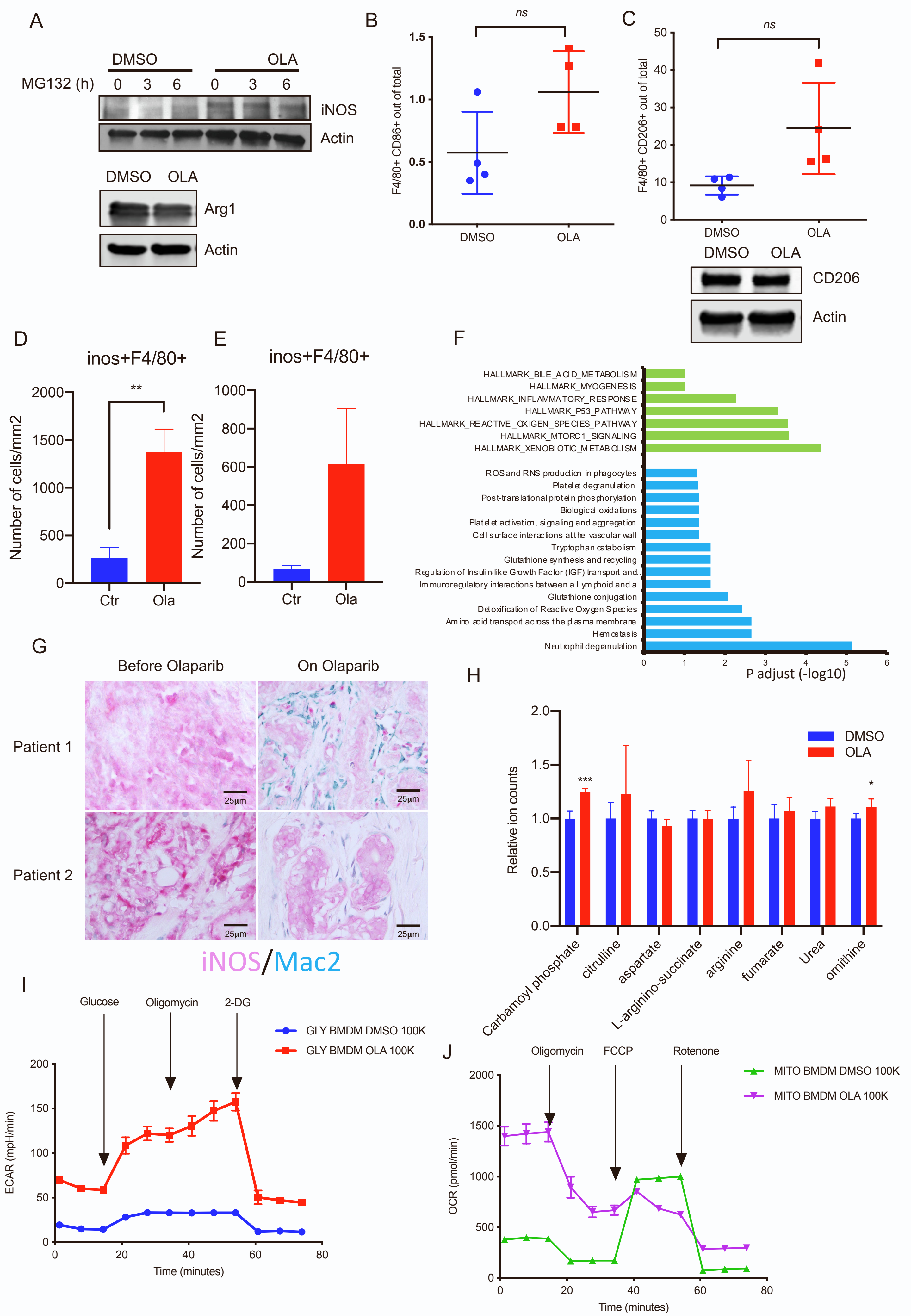

A
DMSO
OLA
MG132 (h)
0 3 6 0 3 6
iNOS
Actin
DMSO OLA
Arg1
Actin
B
ns
F4/80+ CD86+ out of total
DMSO
OLA
C
ns
F4/80+ CD206+ out of total
DMSO
OLA
DMSO OLA
CD206
Actin
D
inos+F4/80+
**
Number of cells/mm2
Ctr
Ola
E
inos+F4/80+
Number of cells/mm2
Ctr
Ola
F
HALLMARK_BILE_ACID_METABOLISM
HALLMARK_MYOGENESIS
HALLMARK_INFLAMMATORY_RESPONSE
HALLMARK_P53_PATHWAY
HALLMARK_REACTIVE_OXIGEN_SPECIES_PATHWAY
HALLMARK_MTORC1_SIGNALING
HALLMARK_XENOBIOTIC_METABOLISM
ROS and RNS production in phagocytes
Platelet degranulation
Post-translational protein phosphorylation
Biological oxidations
Platelet activation, signaling and aggregation
Cell surface interactions at the vascular wall
Tryptophan catabolism
Glutathione synthesis and recycling
Regulation of Insulin-like Growth Factor (IGF) transport and...
Immunoregulatory interactions between a Lymphoid and a...
Glutathione conjugation
Detoxification of Reactive Oxygen Species
Amino acid transport across the plasma membrane
Hemostasis
Neutrophil degranulation
P adjust (-log10)
G
Before Olaparib
On Olaparib
Patient 1
Patient 2
25µm
iNOS/Mac2
H
DMSO
OLA
Relative ion counts
***
*
Carbamoyl phosphate
citrulline
aspartate
L-arginino-succinate
arginine
fumarate
Urea
ornithine
I
Glucose
Oligomycin
2-DG
GLY BMDM DMSO 100K
GLY BMDM OLA 100K
ECAR (mpH/min)
Time (minutes)
J
Oligomycin
FCCP
Rotenone
MITO BMDM DMSO 100K
MITO BMDM OLA 100K
OCR (pmol/min)
Time (minutes)

Figure S3. PARP-inhibition induces a pro-inflammatory macrophage phenotype. Related to Figure 3

(A) Immunoblot of lysates from BMDMs treated with MG132 for 3h or 6h and analyzed for iNOS (upper panel). MG132 was used to allow for an accumulation of the INOS protein which was otherwise hard to detect. Arginase protein levels were determined by immunoblot in the same cells (bottom panel).

(B-C) BMDMs were derived from mouse bone marrow (4 donor mice), cultured, differentiated and treated for 6 days as in Fig. S2A and subjected to flow cytometry. Gating strategy as in Fig. S2E, except that cells were stained with F4/80, CD86 or CD206. The percentage of F4/80 and CD86 dual-positive cells (B) and F4/80 and CD206 dual-positive cells (C) was compared between control and Olaparib-treated cultures. Data are presented as mean +/- SD for the respective population and compared using an unpaired t-test.

(D-E) FVT2 (K14cre Brcaf/f p53f/f) tumors (D) or p53T (K14cre Brca wt/wt p53wt/f) tumors (E) were implanted in FVB mice (n=8). Mice were treated with Olaparib for 10 days. FFPE of tumors were stained with iNOS and F4/80 antibody and DAPI using Akoya Opal multiplex IHC kit. Numbers of iNOS and F4/80 double positive cells were analyzed.

(F) Corresponding to Fig. 3D. BMDMs, derived and treated as outlined in Fig.S2A (4 donor mice), were harvested and total RNA extracted from cells and transcriptomic changes were determined via RNA-seq. The most significantly changed pathways among top 500 differentially regulated genes are shown, with Hallmark in green and Reactome pathways in blue.

(G) Double stain IHC of human biopsy before or after olaparib therapy from patients participating in the PETREMAC study. Proteins stained were shown as indicated.

(H) BMDMs (4 donor mice) were harvested and metabolites extracted from cells and determined via Mass Spectrometry. The metabolites in the urea cycle are shown. Data are presented as mean +/- SD of quadruplicates. Significance analyzed using an unpaired t-test.

(I-J) Repeat of Fig. 3 F, G. $1x10^5$ of BMDM were harvested and seeded in seahorse assay 24 well plate and cultured overnight. Extracellular acidification rate (I) and oxygen consumption rate (J) were determined using a Seahorse analyzer. Data presented as mean +/- SD of 5 (I) or 6 (J) replicates obtained from 3 donor mice per experiment, representative of duplicate experiment.

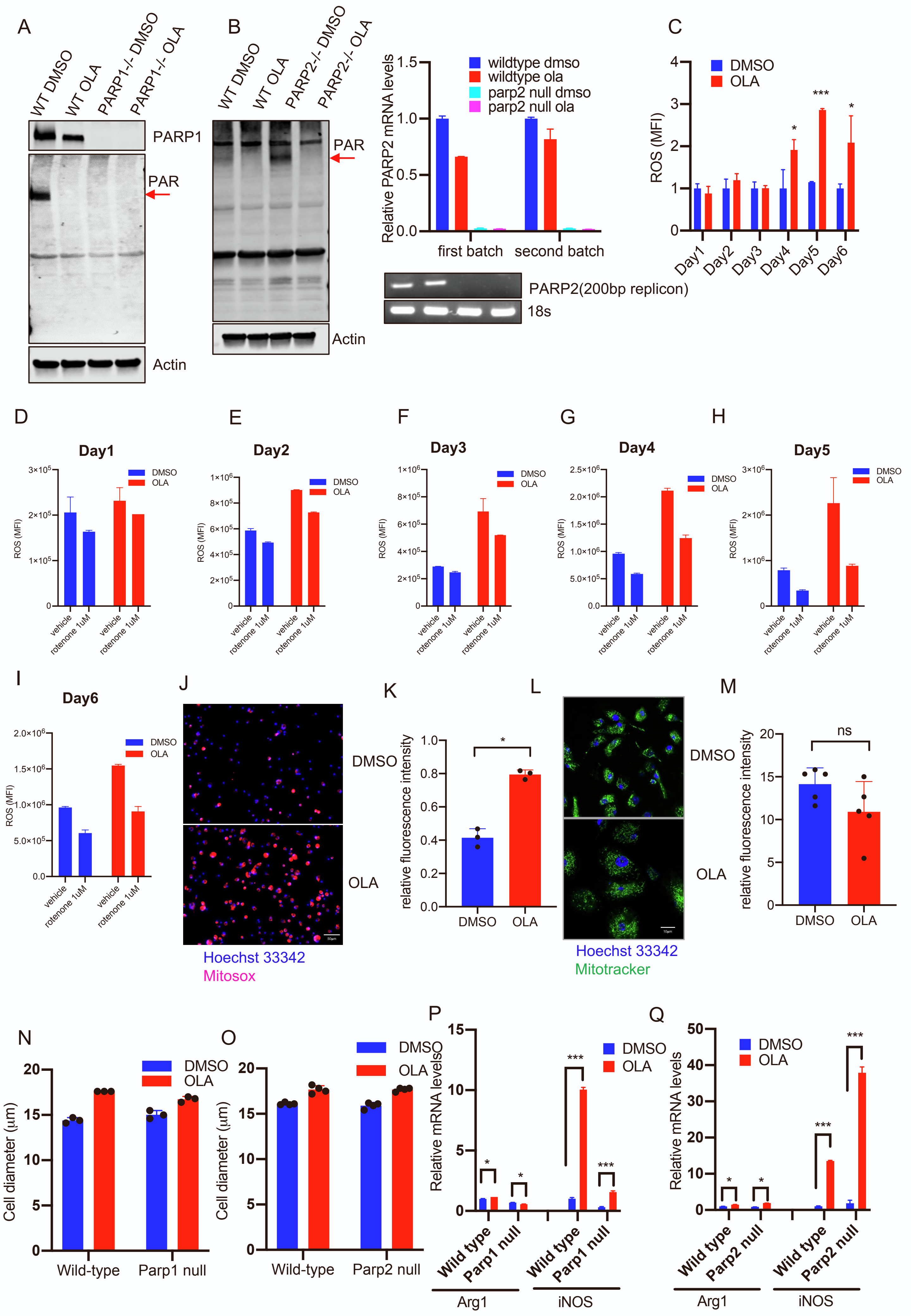
A
WT DMSO
WT OLA
PARP1-/- DMSO
PARP1-/- OLA
PARP1
PAR
Actin
B
WT DMSO
WT OLA
PARP2-/- DMSO
PARP2-/- OLA
PAR
Actin
Relative PARP2 mRNA levels
wildtype dmso
wildtype ola
parp2 null dmso
parp2 null ola
first batch
second batch
PARP2(200bp replicon)
18s
C
ROS (MFI)
DMSO
OLA
Day1
Day2
Day3
Day4
Day5
Day6
D
Day1
E
Day2
F
Day3
G
Day4
H
Day5
I
Day6
ROS (MFI)
vehicle
rotenone 1uM
J
DMSO
OLA
Hoechst 33342
Mitosox
K
relative fluorescence intensity
L
DMSO
OLA
Hoechst 33342
Mitotracker
M
relative fluorescence intensity
ns
N
Cell diameter (μm)
Wild-type
Parp1 null
O
Cell diameter (μm)
Wild-type
Parp2 null
P
Relative mRNA levels
Wild type
Parp1 null
Arg1
iNOS
Q
Relative mRNA levels
Wild type
Parp2 null
Arg1
iNOS

Figure S4. PARP-inhibition facilitates ROS production via RET and induces macrophage reprogramming independent of PARP1/2. Related to Figure 4

(A-B) PARP1/2 and parylation were determined in BMDMs from wildtype (2 donor mice), PARP1 (A, 2 donor mice) and PARP2 null mice (B, 2 donor mice). Macrophages were isolated, differentiated as described in Fig. 3A, as indicated and subjected to immunoblotting or real-time PCR.

(C) BMDM cells (3 donor mice per time point) were treated with olaparib for different days as indicated. Cells were harvested and stained with DCFDA and ROS content was determined via flow cytometry. Data represent mean +/- SD of triplicate cultures for each time point. Unpaired t-test.

(D-I) Reversal of Olaparib-induced ROS with Rotenone. BMDM cells (3 donor mice per time point) were treated with rotenone as indicated. Cells were harvested at different days of olaparib treatment and stained with DCFDA and ROS content was determined via flow cytometry. Data represent mean +/- SD of triplicate cultures for each time point.

(J-M) Mouse bone marrow cells were cultured in the presence of IL4, IL10 and MCSF and olaparib or DMSO. Cells were harvested on day 6. Cells were stained with mitosox (J) or mitotracker (L) and DAPI and the intensity of Mitosox (K) or mitotracker (M) fluorescence were calculated. Significance was determined using an unpaired student's t-test.

(N-Q) BMDMs from wildtype (3 donor mice) and PARP1/2 null (3 donor mice for each genotype) mice were analyzed for cell diameter (N-O) and Arg1 and iNOS mRNA levels (P-Q). Data represent mean +/- SD of quadruplicate cultures for cell diameter in N and triplicate cultures cell diameter in O and for mRNA determination (P,Q). Significance was determined using an unpaired student's t-test.

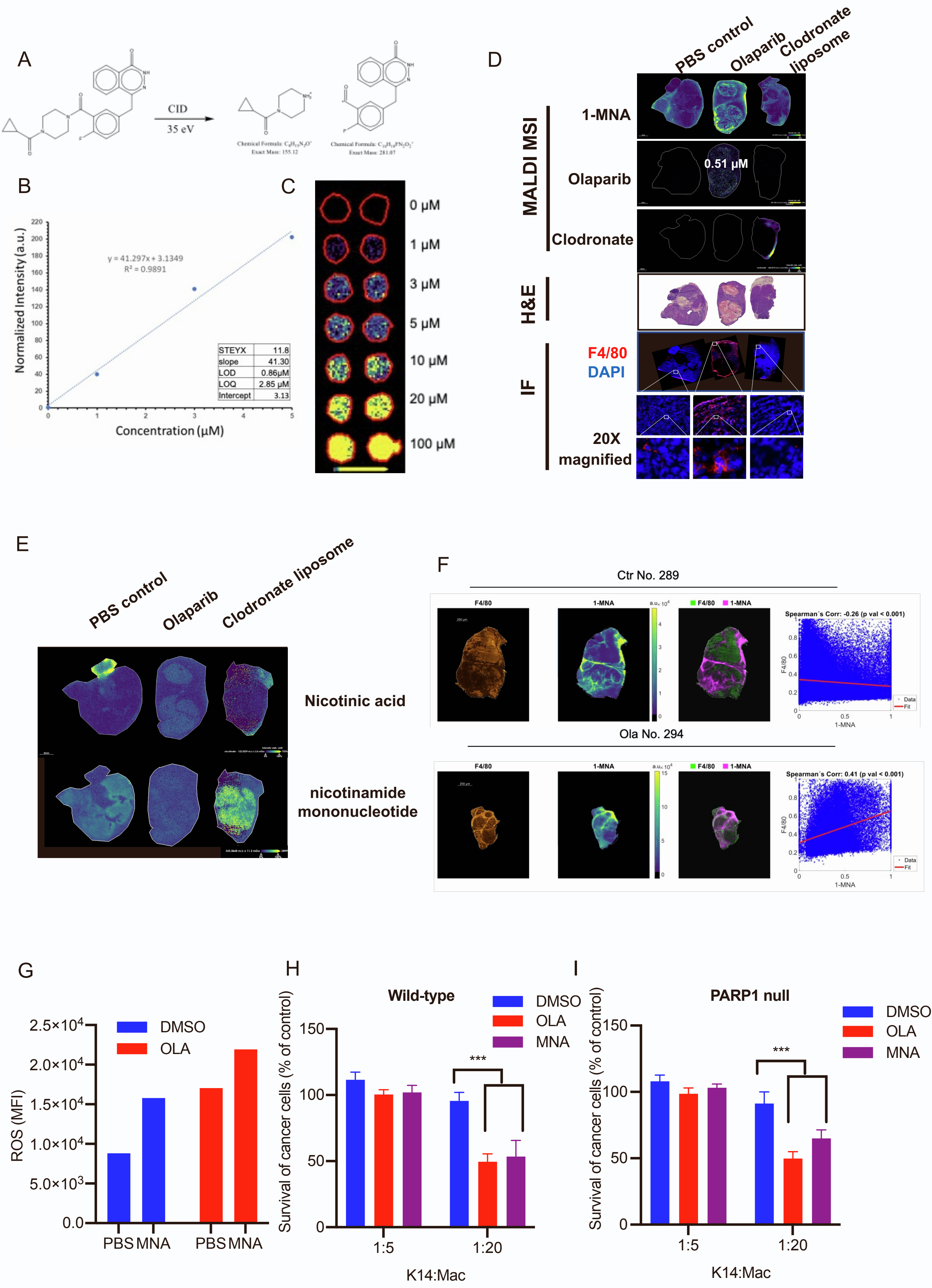
A
CID
35 eV
B
Normalized Intensity (a.u.)
Concentration (μM)
y = 41.297x + 3.1349
R² = 0.9891
STEYX 11.8
slope 41.30
LOD 0.86μM
LOQ 2.85 μM
Intercept 3.13
C
0 μM
1 μM
3 μM
5 μM
10 μM
20 μM
100 μM
D
PBS control
Olaparib
Clodronate liposome
MALDI MSI
1-MNA
Olaparib
0.51 μM
Clodronate
H&E
IF
F4/80
DAPI
20X magnified
E
PBS control
Olaparib
Clodronate liposome
Nicotinic acid
nicotinamide mononucleotide
F
Ctr No. 289
F4/80
1-MNA
Spearman´s Corr: -0.26 (p val < 0.001)
Ola No. 294
Spearman´s Corr: 0.41 (p val < 0.001)
Data
Fit
G
ROS (MFI)
DMSO
OLA
PBS MNA PBS MNA
H
Wild-type
Survival of cancer cells (% of control)
DMSO
OLA
MNA
***
1:5
1:20
K14:Mac
I
PARP1 null
Survival of cancer cells (% of control)
DMSO
OLA
MNA
***
1:5
1:20
K14:Mac

Figure S5. PARP-inhibition leads to a shift in nicotinamide metabolism including accumulation of Methyl-nicotinamide (MNA) which can phenocopy olaparib's effect on BMDMs. Related to Figure 5

(A) Chemical structure of olaparib precursor and product ions that were used for tissue quantification.

(B) Calibration curve for olaparib MALDI MSI quantification with R2 = 0.9891.

(C) Tissue mimetic analysis of olaparib ranging from 0.0-100 µM.

(D-E) Tumor-bearing mice were treated with olaparib or clodronate liposome or PBS control. Upon euthanasia, tumors were frozen in liquid nitrogen and frozen sections subjected to MALDI MSI. H&E stain to determine the morphology of the tumors, Immunofluorescence stain of F4/80 and DAPI to identify macrophages in the tumors (D), NAD salvage synthesis metabolites were detected, related to Fig 5A (E).

(F) The correlation of methyl-nicotinamide and macrophages in consecutive sections were calculated in Ctr (NO. 289) and Ola (NO. 294) tumors.

(G) BMDMs (1 WT donor mouse) were treated with MNA alone or together with olaparib. Cells were harvested and ROS measured, results from a single experiment.

(H-I) Mouse BMDM harvested from PARP1 null mice and wild-type controls (6 donor mice for each genotype) were cultured in the presence of cytokines and with olaparib or MNA or DMSO for 6 days. Cells were harvested and co-cultured with K14-GFP at ratios as indicated. Phagocytosis of macrophages from wildtype (H) or PARP1 null (I) mice was analyzed via BLI assay. Data represent mean +/- SD of six replicate cultures for each group. Significance was determined using an unpaired student's t-test.

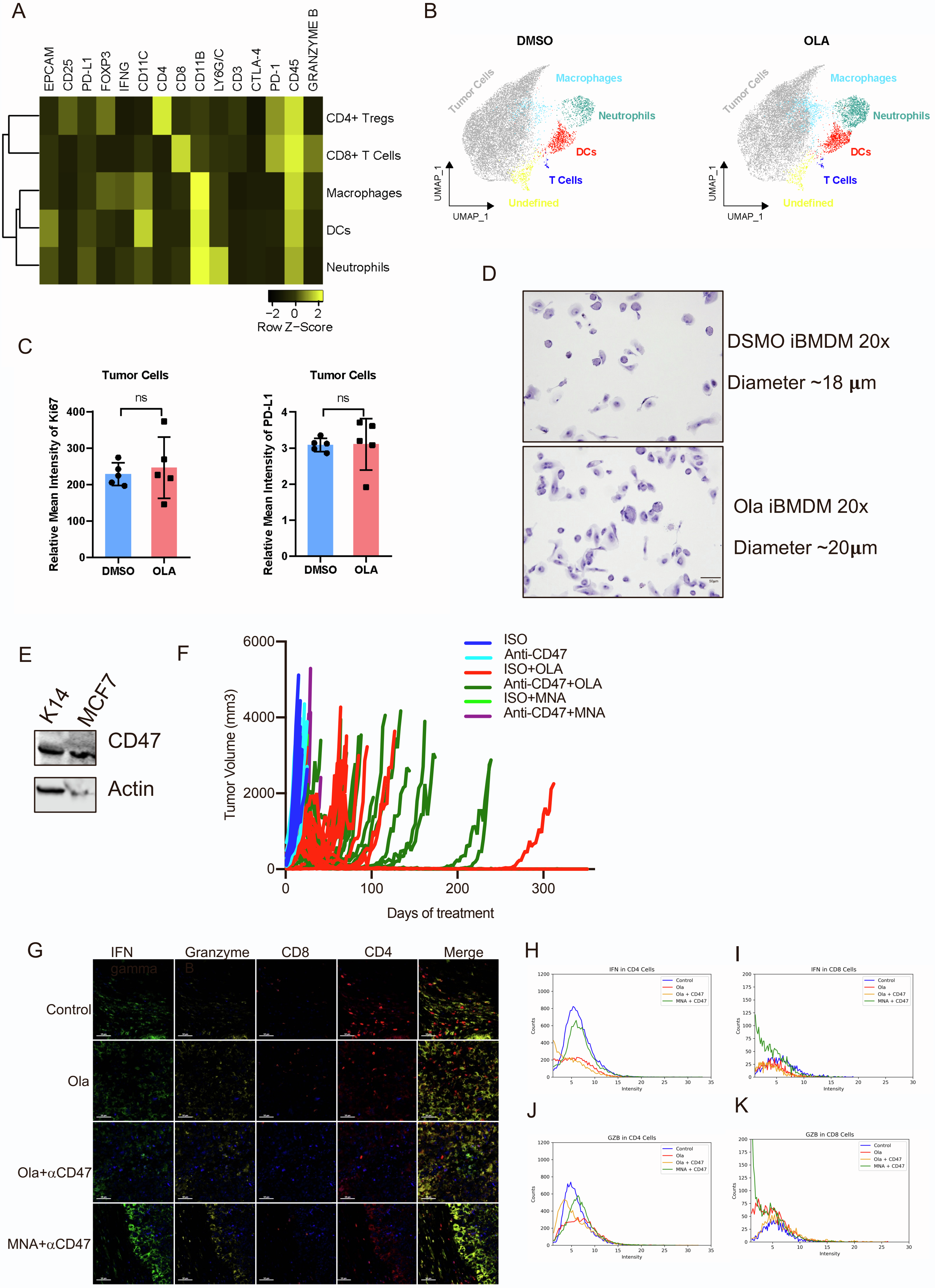
A
B
DMSO
OLA
Tumor Cells
Macrophages
Neutrophils
DCs
T Cells
Undefined
UMAP_1
CD4+ Tregs
CD8+ T Cells
Macrophages
DCs
Neutrophils
Row Z-Score
C
Tumor Cells
ns
Relative Mean Intensity of Ki67
Relative Mean Intensity of PD-L1
DMSO
OLA
D
DSMO iBMDM 20x
Diameter ~18 µm
Ola iBMDM 20x
Diameter ~20µm
E
K14
MCF7
CD47
Actin
F
ISO
Anti-CD47
ISO+OLA
Anti-CD47+OLA
ISO+MNA
Anti-CD47+MNA
Tumor Volume (mm3)
Days of treatment
G
IFN gamma
Granzyme B
CD8
CD4
Merge
Control
Ola
Ola+αCD47
MNA+αCD47
H
IFN in CD4 Cells
I
IFN in CD8 Cells
J
GZB in CD4 Cells
K
GZB in CD8 Cells
Counts
Intensity
Control
Ola
Ola + CD47
MNA + CD47

Figure. S6 Olaparib-treated macrophages increase macrophage recruitment and enhance CD8 T cell cytotoxicity in the TME. Related to Figure 6

(A-C) Antigenic profile of cell populations determined by CyTOF (A). Uniform Manifold Approximation and Projection (UMAP) separation of observed cell populations in two dimensions (B) and Ki67 and PD-L1 levels in tumor cells (C).

D) H&E stain of iBMDM treated with olaparib or DMSO.

(E) CD47 expression in K14-cells was determined via western blot.

(F) Tumor growth curves, related to Fig 6 J-K.

(G-K) K14 (K14cre Brcaf/f p53f/f) tumors were implanted in recipient mice (n=6). Mice were treated with Olaparib alone or together with CD47 or MNA together with CD47 for 10 days. Tumors were stained with antibodies and DAPI as indicated using OPAL multiplex IHC kit. G, Representative images were shown. H-K, statistical analysis of intensity of IFN in CD4+ (H) or CD8+ (I) T cells and Granzyme B in CD4+ (J) or CD8+ (K) cells were analyzed.

Table S1. TM-sprayer MALDI spray parameters (related to Figure 5)

Matrix	Flow rate (mL/min)	Temperature (°C)	# passes	Velocity (mm/min)	Track spacing (mm)	N_2 gas (psi)
DHB	0.18	75	2	1200	2	10
CHCA	0.17	75	2	1200	2	10
DAN-HCl	0.09	75	4	1200	2	10

Table S2. Down-regulated pathways among overlap regulated genes (related to Figure 5)

Term	Count	Fold Enrichment	Bonferroni	Benjamini	FDR
rRNA processing	49	2.599660038	4.39E-06	4.39E-06	4.37E-06
positive regulation of transcription from RNA polymerase II promoter	133	1.539277372	0.00168535	8.43E-04	8.40E-04
mRNA splicing, via spliceosome	44	2.250266494	0.00266017	8.88E-04	8.84E-04
positive regulation of transcription, DNA-templated	79	1.741622849	0.00572752	0.001436	0.00142991
regulation of transcription from RNA polymerase II promoter	67	1.724925986	0.05440734	0.0111886	0.01114116
RNA splicing, via transesterification reactions	11	4.995591616	0.10359615	0.01822714	0.01814985
viral process	49	1.860626248	0.129511	0.01981398	0.01972997
transcription from RNA polymerase II promoter	73	1.615621956	0.18498101	0.02528174	0.02517454
maturation of SSU-rRNA from tricistronic rRNA transcript (SSU-rRNA, 5.8S rRNA, LSU-rRNA)	12	4.257606491	0.20351083	0.02528174	0.02517454
response to endoplasmic reticulum stress	19	2.876249718	0.24758083	0.02844522	0.0283246
RNA processing	22	2.575047225	0.30955129	0.03367249	0.03352971
histone acetylation	12	4.00715905	0.34815509	0.03566059	0.03550938
IRE1-mediated unfolded protein response	16	3.078947067	0.43164637	0.04345953	0.04327525

Table S3. Primers for qPCR (related to star methods)

Gene name	Forward Primer	Reverse Primer
mArg1	CTTGGGAAGACAGCAGAGGA	AGCAAGCCAAGGTTAAAGCC
mPparg	GGAATCAGCTCTGTGGACCT	GTGGAGCAGAAATGCTGGAG
mPpard	CCGCATGAAGCTCGAGTATG	ACTGACACTTGTTGCGGTTC
mPgc1b	GAACTGTGACCCTACCCACA	TTTACAGGACGCCAGGTCTT
mLpl	GGCCCAGCAACATTATCCAG	ACCCTAAGAGGTGGACGTTG
mCd36	GCCAAGCTATTGCGACATGA	GGCATTGGCTGGAAGAACAA
mAcadm	GAAAGCTGCTAGTGGAGCAC	CTGGTAACTGAGCCTAGCGA
mAcadl	TCCTCACCACACAGAATGGG	GCCATGTTTCTCTGCGATGT
mRetnla	TGGGAGATCCAGAGTGGAGA	TCTTAGGACAGTTGGCAGCA
mYm1	ATGAAGGAGCCACTGAGGTC	CCACGGCACCTCCTAAATTG
mMgl1	ACTGTGCCCACATCACTACA	CTTGGCCAGCTTCATCTCAC
mNos2	GTTCTCAGCCCAACAATACAAGA	GTGGACGGGTCGATGTCAC
mIl6	CTGCAAGAGACTTCCATCCAG	AGTGGTATAGACAGGTCTGTTGG
mTnfa	CTCATGCACCACCATCAAGG	ACCTGACCACTCTCCCTTTG
mIl1b	GAAATGCCACCTTTTGACAGTG	TGGATGCTCTCATCAGGACAG
m18S	GTAACCCGTTGAACCCCATT	CCATCCAATCGGTAGTAGCG
mParp2	ACCAAGGGGATGGGAAAG	TCACCATAGCTGCAGGAA
